# ACOD1, rather than itaconate, facilitates p62‐mediated activation of Nrf2 in microglia post spinal cord contusion

**DOI:** 10.1002/ctm2.1661

**Published:** 2024-04-22

**Authors:** Zhanyang Qian, Mingjie Xia, Tianyu Zhao, You Li, Guangshen Li, Yanan Zhang, Haijun Li, Lei Yang

**Affiliations:** ^1^ Department of Orthopedics Taizhou School of Clinical Medicine Taizhou People's Hospital of Nanjing Medical University, Nanjing Medical University Taizhou China; ^2^ Department of Spine Surgery Nantong First People's Hospital The Second Affiliated Hospital of Nantong University Nantong China; ^3^ Postgraduate School Dalian Medical University Dalian China; ^4^ Department of Trauma and Reconstructive Surgery RWTH Aachen University Hospital Aachen Germany

**Keywords:** aconitate decarboxylase 1, itaconate, neuroinflammation, Nrf2, p62, spinal cord injury

## Abstract

**Background:**

Spinal cord injury (SCI)‐induced neuroinflammation and oxidative stress (OS) are crucial events causing neurological dysfunction. Aconitate decarboxylase 1 (ACOD1) and its metabolite itaconate (Ita) inhibit inflammation and OS by promoting alkylation of Keap1 to induce Nrf2 expression; however, it is unclear whether there is another pathway regulating their effects in inflammation‐activated microglia after SCI.

**Methods:**

Adult male C57BL/6 *ACOD1^−/−^* mice and their wild‐type (WT) littermates were subjected to a moderate thoracic spinal cord contusion. The degree of neuroinflammation and OS in the injured spinal cord were assessed using qPCR, western blot, flow cytometry, immunofluorescence, and trans‐well assay. We then employed immunoprecipitation‐western blot, chromatin immunoprecipitation (ChIP)‐PCR, dual‐luciferase assay, and immunofluorescence‐confocal imaging to examine the molecular mechanisms of ACOD1. Finally, the locomotor function was evaluated with the Basso Mouse Scale and footprint assay.

**Results:**

Both in vitro and in vivo, microglia with transcriptional blockage of ACOD1 exhibited more severe levels of neuroinflammation and OS, in which the expression of p62/Keap1/Nrf2 was down‐regulated. Furthermore, silencing ACOD1 exacerbated neurological dysfunction in SCI mice. Administration of exogenous Ita or 4‐octyl itaconate reduced p62 phosphorylation. Besides, ACOD1 was capable of interacting with phosphorylated p62 to enhance Nrf2 activation, which in turn further promoted transcription of ACOD1.

**Conclusions:**

Here, we identified an unreported ACOD1‐p62‐Nrf2‐ACOD1 feedback loop exerting anti‐inflammatory and anti‐OS in inflammatory microglia, and demonstrated the neuroprotective role of ACOD1 after SCI, which was different from that of endogenous and exogenous Ita. The present study extends the functions of ACOD1 and uncovers marked property differences between endogenous and exogenous Ita.

**Key points:**

- ACOD1 attenuated neuroinflammation and oxidative stress after spinal cord injury.
- ACOD1, not itaconate, interacted with p‐p62 to facilitate Nrf2 expression and nuclear translocation.
- Nrf2 was capable of promoting ACOD1 transcription in microglia.

## Graphical abstract

An unreported anti‐inflammatory/oxidative feedback loop between ACOD1 and Nrf2 regulated by phosphorylation of p62 during neuroinflammation after SCI.

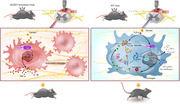

## 1 INTRODUCTION

Traumatic spinal cord injury (SCI) causes acute mechanical contusion of neural tissue and induces prominent neuroinflammation, a characteristic that is evident in brain injury and other acute neurological diseases.^1^, ^2^ Neuroinflammation and oxidative stress (OS) following SCI trigger secondary neuropathies such as glial accumulation, neuronal loss, and demyelination that exacerbate neurological dysfunction and functional disorder.^3^, ^4^, ^5^ Microglia are the resident immune cell of the central nervous system and become activated immediately after a trauma, and have been recognised as one of the key mediators of neuroinflammation in the acute phase of SCI.^6^, ^7^ Moreover, microglia actively produce multiple oxidisers including nitric oxide (NO) and reactive oxygen species (ROS) that contribute to neurotoxicity.^8^, ^9^, ^10^ Therefore, coordinate metabolic changes in microglia are crucial for the resolution of neuroinflammation, neuronal protection and histological remodelling.^11^, ^12^

Aconitate decarboxylase 1 (ACOD1), also known as IRG1, regulates the immune phenotype of macrophages to reduce tissue damage and promote repair after pathogenic insult.^13^, ^14^, ^15^ Increased AOCD1 expression in lipopolysaccharide (LPS)‐treated macrophages has been shown to promote the conversion of itaconate (Ita) from aconitate, the latter of which is a typical product of metabolic reprogramming that further inhibits generation of succinate dehydrogenase.^16^, ^17^, ^18^ Ita has been viewed as an anti‐inflammatory because it has been shown to induce expression of Nrf2, a transcription factor with ant‐inflammatory and OS properties, in LPS‐stimulated murine and human macrophages.^19^, ^20^ Through protein modification of Keap1 via alkylation of its cysteine residues, Ita promotes Nrf2 expression, which enhances transcription of downstream genes that exert anti‐inflammatory/oxidative functions.^19^, ^21^ Because of this property, Ita and its derivative 4‐octyl itaconate (4‐OI) have been suggested as a potential target for the therapy of various diseases including liver injury, neuronal infection and pulmonary fibrosis.^22^, ^23^, ^24^ Our recent study confirmed that the expression of ACOD1 was markedly increased in inflammatory microglia after LPS stimulation.^25^ Besides, 4‐OI also plays an anti‐inflammatory role by inducing Nrf2 expression after SCI.^26^ However, evidence has shown that the anti‐inflammatory mechanism of 4‐OI is different from that of ACOD1/Ita.^27^ Furthermore, it is unknown whether AOCD1 regulates Nrf2 by other pathways that are independent of Keap1 alkylation, and whether Nrf2, as an anti‐inflammatory transcription factor, further promotes the transcription of ACOD1 in microglia under a neuroinflammatory environment.

In the present study, we found that ACOD1 promotes Nrf2 activation via increasing p62 phosphorylation‐induced Keap1 degradation, and Nrf2 further enhances the transcription of ACOD1 in microglia during neuroinflammation. Treatment with Ita or 4‐OI not only decreased p62 phosphorylation, which was consistent with the effect of silencing ACOD1 in microglia. Mice with knockdown of AOCD1 were found to have defective pathologies (glial scar formation, demyelination, and axonal degeneration) and increased neural dysfunction during the progression of SCI.

## 2 METHODS

### 2.1 Bioinformatic analysis

#### 2.1.1 Data sources and preprocessing

We downloaded acute SCI mouse RNA‐sequencing (seq) data from the GEO database (https://www.ncbi.nlm.nih.gov/geo/) and selected the GSE5296 gene microarray dataset (Mus musculus, T8 SCI). The dataset included data from six sham mice (the control group) and six mice 24 h after SCI (the SCI group). Data from microarray experiments were normalised using the Bioconductor package limma.^28^ The Uniform Manifold Approximation and Projection (UMAP) algorithm was used to confirm the segregation of groups control and SCI based on normalised gene chip data.

#### 2.1.2 Differential gene expression analysis

To determine the expression of genes in SCI, we performed differentially expressed genes (DEGs) analysis using the R package limma and visualised using the ggplot2 package.^29^ After multiple corrections using the Benjamini and Hochberg method, we set a cutoff at a *Q*‐value of < .05 and an absolute log fold change ≥1.0 to ensure the comprehensiveness of the differential expression analysis.

#### 2.1.3 Gene set variation analysis

Gene set variation analysis (GSVA) is an enrichment algorithm that estimates changes in pathway and the biological process activity in samples of expression datasets. To determine whether there were differences between different groups regarding biological processes, we performed GSVA using the gene expression profiling dataset of ASCI mice. Using the annotation catalog (msigdb.v7.4.symbols.gmt) from the MSigDB database,^30^ we performed GSVA using the R package GSVA,^31^ and employed linear fitting and Bayesian network algorithms to determine the differences between the SCI and control groups in the relevant GSVA pathways. A *P*‐value threshold of.05 was used to determine statistical significance.

#### 2.1.4 Trend clustering and functional enrichment analyses

To analyse the potential subtypes among the DEGs of SCI mice, we first organised the expression matrix of DEGs, then compressed the data distribution using normalisation, and finally performed trend clustering analysis using the R package Mfuzz.^32^

DEGs annotation using gene ontology (GO) and kyoto encyclopedia of genes and genomes (KEGG) pathway enrichment analyses was performed using the clusterProfiler package^33^ in R. Statistical significance was determined using a false discovery rate of.05.

#### 2.1.5 Statistical analysis

Data processing and analysis were conducted using the R software (Version 4.2.0). When comparing two continuous variable groups, an independent Student's *t*‐test was used to estimate the statistical significance of normally distributed variables, and Mann–Whitney *U*‐tests were used to examine differences between non‐normally distributed variables. Statistical significance of the two categorical variable groups was determined using the chi‐square or Fisher's exact test. Spearman correlation analysis was used to measure the correlation coefficients of different genes. The statistical *p*‐values were two‐tailed, and statistical significance was set at *P* < .05.

### 2.2 Animals

The project was approved by the Ethics Committee of Taizhou People's Hospital of Nanjing Medical University and the procedure was performed in line with the guidelines of the National Institutes of Health Animal Laboratory Animal Care and Use Guidelines. We obtained ACOD1 knockout mice (KO, *ACOD1* ^−/−^) (C57BL/6N background) mice from Cyagen Biosciences (Guangzhou, China). The generation of the knockout strain was, briefly, as follows: a synthetic gRNA targeting the ACOD1 gene sequence between exon 2 and 4 (Figure S5A) and Cisper‐Cas9 nickase were injected in wild‐type (WT) C57BL/6J mouse oosperms, which were then transplanted into the uterus of surrogate mother mice. *ACOD1* ^+/−^ mice were then mated with each other to acquire *ACOD1* ^−/−^ mice (Figure S5B). Adult WT C57BL/6J mice served as the control.

### 2.3 Spinal cord injury

C57BL/6J mice (male, 8‐week‐old, weighted 20 g) were employed to establish a traumatic SCI model, as previously described.^34^ Briefly, mice were anesthetised by ketamine/xylazine injection and a moderate contusion created (5 g × 5 cm) at the T10 level using a spinal cord impactor (68097, RWD, Shenzhen, China). After, the wound was sutured and the mice placed in a temperature‐controlled cage for recovery. Motor functional recovery was assessed with an open‐field basso mouse scale (BMS) and footprint assay as previously described.^25^, ^35^ Two investigators scored the locomotor ability of each mouse in a blinded manner.

### 2.4 Preparation of neural debris

Adult WT C57BL/6J mice were anaesthetised and SCI performed as above. The mice were then sacrificed immediately and approximately 5 mm of T10 spinal cord tissue was removed, homogenised, and centrifugated (14,000 rpm/min, 15 min) at 4°C to collect neural debris. The resultant pellet was washed twice with phosphate buffer saline (PBS) at 14,000 rpm for 15 min each, resuspended to a final concentration of 100 mg/mL with PBS, and stored at −80°C.

### 2.5 Isolation and culture of glial cells

Primary microglia and astrocytes were isolated as described previously.^25^ Briefly, glial cells were isolated from the cortices of 3‐day neonatal mice by digestion with.25% trypsin (NCM Biotech, Suzhou, China). After culturing for 15−20 days, microglia were collected by shaking at 200 rpm for 4 h and subsequently seeded in Dulbecco's modified eagle medium (DMEM, KeyGEN, Nanjing, China) supplemented 10% fetal bovine serum (FBS, Gibco, Grand Island, NY, USA).

After culturing for 3 days, astrocytes were isolated by shaking for 2 h. The cells were then incubated with.05% trypsin for 15 min at 37°C to remove oligodendrocytes. After neutralisation with complete DMEM, the astrocytes were routinely subcultured. The purity of primary microglia and astrocyte cultures was > 90%.

### 2.6 Cell transfection

Microglia were transfected with lentivirus shRNA‐negative control (LV‐NC), lentivirus shRNA‐ACOD1 (LV‐ACOD1i), or lentivirus ACOD1 (LV‐ACOD1) at 10^7^ TU/mL supplemented 1 × HitransG A (GeneChem, Shanghai, China) for 12 h. The medium was then exchanged and the cells cultured for a further 60 h. Meanwhile, HEK‐293T cells were transfected with plasmid loading siRNA‐negative control (si‐NC), siRNA‐ACOD1 (si‐ACOD1) and ubiquitin‐Flag, Nrf2‐6his, ubiquitin‐K63‐HA, and ubiquitin‐K48‐MYC (GeneChem) at 1.5 μg/mL with Lipo293 Transfection Reagent (C0521, Beyotime) for 48 h. BV2 microglia were transfected with plasmid loading p‐p62 or mutation of p‐p62 (both from GeneChem) at 3 mg with the above reagent for 48 h.

### 2.7 Debris‐induced microglial neuroinflammation

A post‐SCI neuroinflammation model in vitro was established using neural debris (1 or 2 mg/mL) to stimulate microglia for 24 h. Depending on the experimental paradigm, microglia were pretreated with Ita (10 mM, MedChemExpress, NJ, USA) or 4‐Octyl Itaconate (4‐OI, 125 μM, MedChemExpress) dissolved in.1% DMSO for 3 h; or the Nrf2 activator NK‐252 (10 μM, MedChemExpress) or Nrf2 inhibitor Nrf2‐IN‐1 (5 μM, MedChemExpress) dissolved in.1% DMSO for 48 h. LPS (1 μg/mL, Sigma–Aldrich, St. Louis, MO, USA) dissolved in PBS was used to treat microglia or 293T cells for a further 24 h.

### 2.8 qPCR

Total RNA was extracted from cells using TRIzol reagent (YIFEIXUE BioTech, Nanjing, China). The concentration and purity of RNA were determined using a NanoDrop‐2000 spectrophotometer (Thermo Fisher Scientific, Waltham, MA, USA) at 260/280 nm. Reverse transcription of RNA using a YfxScript 1st Strand cDNA Synthesis System (YIFEIXUE BioTech) in accordance with the manufacturer's instruction, followed by its quantification using a 2 × SYBR Green Fast qPCR Master Mix (YIFEIXUE BioTech). The target genes were normalised to *GAPDH* using the 2^Δ^Ct method. The primers are listed in Table 1.

**TABLE 1:** Primers of interest for qPCR.

Gene Name	Forward Sequence (5′−3′)	Reverse Sequence (5′−3′)
ACOD1	GGCACAGAAGTGTTCCATAAAGT	GAGGCAGGGCTTCCGATAG
GAPDH	AGGTCGGTGTGAACGGATTTG	GGGGTCGTTGATGGCAACA
IL‐1β	GAAATGCCACCTTTTGACAGTG	TGGATGCTCTCATCAGGACAG
IL‐6	CTGCAAGAGACTTCCATCCAG	AGTGGTATAGACAGGTCTGTTGG
iNOS	GTTCTCAGCCCAACAATACAAGA	GTGGACGGGTCGATGTCAC
TNF‐α	CAGGCGGTGCCTATGTCTC	CGATCACCCCGAAGTTCAGTAG
NOX‐1	CCTGATTCCTGTGTGTCGAAA	TTGGCTTCTTCTGTAGCGTTC
NOX‐4	TGCCTGCTCATTTGGCTGT	CCGGCACATAGGTAAAAGGATG
HO‐1	AGGTACACATCCAAGCCGAGA	CATCACCAGCTTAAAGCCTTCT

### 2.9 Western blot

Total protein and nuclear protein were extracted using a Total Protein Extraction Kit and Nuclear Protein Extraction Kit (both from KeyGEN), respectively, according to the manufacturer's instructions. Protein concentrations were next determined using an Enhanced BCA Protein Quantitation Kit (Beyotime, Shanghai, China), and an equivalent concentration of protein was used to perform the western blot assay. Membranes were first probed with the specific primary antibodies overnight at 4°C and then incubated with HRP‐labelled secondary antibodies at room temperature for 1 h, after which the protein expression was visualised using a G: Box Chemiluminescence Imaging System (Syngene, Cambridge, UK). Protein quantification and analysis were performed using ImageJ software (NIH, NY, USA). Full antibody information is listed in Table 2.

**TABLE 2:** Antibodies of interest in the study.

Antibodies Name #Cat. No.	Source	Species	Application	Dilution rate
Anti‐iNOS antibody #ab15323	Abcam	Rb	WB	1:250
NRF2, NFE2L2 Polyclonal antibody #16396‐1‐AP	Proteintech	Rb	WB	1:1000
Anti‐IRG1 antibody #ab222411	Abcam	Rb	WB	1:1000
HO‐1 (E9H3A) Rabbit mAb (Mouse Specific) #86806	CST	Ms	WB	1:1000
Phospho‐SQSTM1/p62 (Ser349) (E7M1A) Rabbit mAb #16177	CST	Rb	WB	1:1000
SQSTM1/p62 (D6M5X) Rabbit mAb (Rodent Specific) #23214	CST	Rb	WB	1:1000
KEAP1 Polyclonal antibody #10503‐2‐AP	Proteintech	Rb	WB	1:1000
Anti‐NOX1 antibody #ab131088	Abcam	Rb	WB	1:1000
NADPH oxidase 4/NOX4 Rabbit mAb #48782	SAB	Rb	WB	1:1000
Cox2 (D5H5) XP Rabbit mAb #12282	CST	Rb	WB	1:1000
Anti‐Ubiquitin antibody [EPR8830] #ab134953	Abcam	Rb	WB	1:1000
Anti‐Ubiquitin (linkage‐specific K48) antibody [EP8589] #ab140601	Abcam	Rb	WB	1:1000
Anti‐Ubiquitin (linkage‐specific K63) antibody [EPR8590‐448] #ab179434	Abcam	Rb	WB	1:1000
HRP‐conjugated GAPDH Monoclonal antibody # HRP‐60004	Proteintech	Ms	WB	1:10000
HRP‐conjugated Beta Actin Monoclonal antibody #HRP‐60008	Proteintech	Ms	WB	1:10000
Anti‐Histone H3 antibody #ab1791	Abcam	Rb	WB	1:2000
Goat Anti‐Rabbit IgG Secondary antibody (H+L), HRP #YFSA02	YIFEIXUE BioTech	Goat	WB	1:10000
Goat Anti‐Mouse IgG Secondary antibody (H+L), HRP # YFSA01	YIFEIXUE BioTech	Goat	WB	1:10000
IPKine™ HRP, Mouse Anti‐Rabbit IgG LCS	Abbkine	Ms	WB	1:2000
Anti‐IRG1 antibody #ab222411	Abcam	Rb	IP	1:30
NRF2 (D1Z9C) XP Rabbit mAb #12721	CST	Rb	IP	1:50
Mouse IgG (Sepharose Bead Conjugate) #3420	CST	Ms	IP	1:20
NRF2 (D1Z9C) XP Rabbit mAb #12721	CST	Rb	ChIP	1:100
KEAP1 Polyclonal antibody #10503‐2‐AP	Proteintech	Rb	IF	1:400
NRF2 (D1Z9C) XP® Rabbit mAb #12721	CST	Rb	IF	1:400
Anti‐iNOS antibody (ab15323)	Abcam	Rb	IF	1:100
Anti‐Iba1 antibody [EPR16588] #ab178846	Abcam	Rb	IF	1:500
Anti‐CD68 antibody [EPR23917‐164] #ab283654	Abcam	Rb	IF	1:50
GFAP (GA5) Mouse mAb #3670	CST	Ms	IF	1:600
Neurofilament‐H (RMdO 20) Mouse mAb #2836	CST	Ms	IF	1:400
Anti‐NOX1 antibody #ab131088	Abcam	Rb	IF	1:200
NADPH oxidase 4/NOX4 Rabbit mAb #48782	SAB	Rb	IF	1:200
Aggrecan Polyclonal antibody	Proteintech	Rb	IF	1:400
Alexa Fluor® 647 AffiniPure Fab Fragment Goat Anti‐Rabbit IgG (H+L) #111607003	Jackson ImmunoResearch	Goat	IF	1:500
Alexa Fluor® 594 AffiniPure Fab Fragment Goat Anti‐Rabbit IgG (H+L) #111587003	Jackson ImmunoResearch	Goat	IF	1:500
Alexa Fluor® 488 AffiniPure Fab Fragment Goat Anti‐Rabbit IgG (H+L) #111547003	Jackson ImmunoResearch	Goat	IF	1:500
Alexa Fluor® 594 AffiniPure F(ab')₂ Fragment Goat Anti‐Mouse IgG (H+L) #115586003	Jackson ImmunoResearch	Goat	IF	1:500
Alexa Fluor 488 AffiniPure F(ab')₂ Fragment Goat Anti‐Mouse IgG (H+L) #115546003	Jackson ImmunoResearch	Goat	IF	1:500
Alexa Fluor 647 AffiniPure Fab Fragment Goat Anti‐Mouse IgG (H+L) #115607003	Jackson ImmunoResearch	Goat	IF	1:500

### 2.10 Immunoprecipitation

Microglia were harvested in Immunoprecipitation (IP) lysis buffer containing 10 mM β‐glycerophosphate/pyrophosphate (Beyotime) and 1 × Cocktail (MedChemExpress) and lysed on a rotary table for 30 min at 4°C. The supernatant was collected after centrifugation for 10 min at 12000×*g* and 4°C and 50 μL protein was removed and designated as the input, while the remainder was incubated with the ACOD1 antibody and Protein G SepharoseTM 4 Fast Flow beads (GE Healthcare, Stockholm, Sweden) on a rotary table overnight at 4°C. The beads were washed by centrifugation at 500×g and 4°C, five times for 5 min each, after which 2 × loading buffer was added and the beads boiled twice for 5 min at 100°C.

### 2.11 Oxidisation determination

l ROS levels: ROS levels in microglia were detected using an ROS Assay Kit (YFX0707, YIFEIXUE BioTech). Briefly, microglia were incubated with 10 μmol/L DCFH‐DA for 20 min at 37°C and washed for three times washed with DMEM, after which the cells were subjected to flow cytometry at a wavelength of 488 nm.
l Superoxide anion levels: microglia were washed twice with PBS and incubated with DMEM containing 10 μmol/L DCFH‐DA and 5 μM dihydroethidium (S0063, Beyotime) for 20 min. The cell was then incubated with Hoechst 33342 Staining Solution for Live Cells (C1027, Beyotime) for a further 10 min. Live cell images were subsequently captured with an immunofluorescence (IF) microscope (Leica, Weztlar, Germany).
l MDA assay: microglia were harvested in WB lysis buffer, malondialdehyde (MDA) working buffer (S0131, Beyotime) added, and the cells boiled for 15 min at 100°C. After the cells samples had cooled, they were centrifuged for 10 min at 1000×*g*, and added to each well of a 96‐well plate, after which the MDA content was quantified at 532 nm using a microplate reader (Biotek, VT, USA).
l Glutathione assay: glutathione levels were measured using a Total Glutathione Assay Kit (S0052, Beyotime) according to the manufacturer's instructions. Briefly, S reagent was added to microglial cultures with vortexing to remove proteins. The mixture was then subjected to two rounds of freeze‐thawing in liquid nitrogen followed by 37°C water. The mixture was then centrifuged for 10 min at 10,000×*g* and 4°C. The resultant supernatant was then incubated with glutathione detection working buffer for 5 min, 5 mg/mL NADPH solution added, and glutathione levels detected at an absorbance of 405 nm.

### 2.12 Chromatin immunoprecipitation assay

The upstream 2,000 bp section of the promoter region of ACOD1 from the National Center for Biotechnology Information database, was screened for five putative DNA‐binding sites for Nrf2 using the Jaspar core database (Table 1). The chromatin immunoprecipitation assay (ChIP)‐IT Express Enzymatic Kit (53009, Active Motif, Carlsbad, CA, USA) was utilised to pull down Nrf2 or IgG antibody. The putative AOCD1 DNA‐binding sites for Nrf2 were examined using PCR, followed by digital imaging of agarose gels. The PCR primers were designed by Dr. You Li (Table S1).

### 2.13 Dual‐luciferase assay

BV2 microglial cell line (5 × 10^4^ cells/mL) was cultured with complete DMEM at 37°C. The Lipofectamine 2000 Reagent (11668‐027, Invitrogen, CA, USA) was mixed intensively with the Nrf2 and ACOD1 DNAs for a 15 min incubation, then added in FBS‐free DMEM and treated with the cells for 6 h at 37°C. The cells then were cultured for another 42 h in complete DMEM, harvested by 1 × PLB lysis buffer, and the fluorescence was determined by a Dual‐Luciferase Detection Kit (E1910, Promega, Madison, WI, USA).

### 2.14 Immunofluorescence examination

Spinal cord tissue was collected after the mice had been sacrificed under anaesthesia and subsequently cut into 5 mm paraffin sections for IF staining. After antigen retrieval and immuno‐blocking, sections were probed with the primary antibodies overnight at 4°C, washed with PBS‐Tween 20 (PBST), further incubated with fluorescent secondary antibodies for 1 h, and then counterstained with diaminidne phenylindole DAPI. Staining was viewed under an IF microscope (Leica) or confocal microscope (Nikon, Tokyo, Japan). The indicated antibodies are provided in Table 2.

### 2.15 Trans‐well assay

Astrocytes (2 × 10^5^ cells/insert) suspended in the serum‐free medium were seeded onto the upper chambers of Trans‐well inserts (8 μm pore size, Corning) in a 24‐well plate seeded with microglia. After culturing for 72 h, the unmigrated astrocytes on the insert membrane were wiped using a swab and the migrated astrocytes under the membrane were fixed with 4% paraformaldehyde for 15 min and stained with crystal violet (KGA229, KeyGEN) for 10 min. The cells were then viewed under a bright‐field microscope (Leica).

### 2.16 Statistical analysis

Data from at least three independent experiments are presented as the mean ± standard error margin (SEM). Analysis of more than two groups was performed using one‐way or two‐way *ANOVA* followed by Tukey's post hoc test, and for two groups using unpaired two‐tailed Student's *t*‐tests. The graphs were generated using GraphPad Prism 8.1 software (San Diego, CA, USA). *P*‐values < .05 were deemed to be statistically significant.

## 3 RESULTS

### 3.1 ACOD1 negatively regulates inflammatory and immune‐related functions after spinal cord injury

**FIGURE 1:**
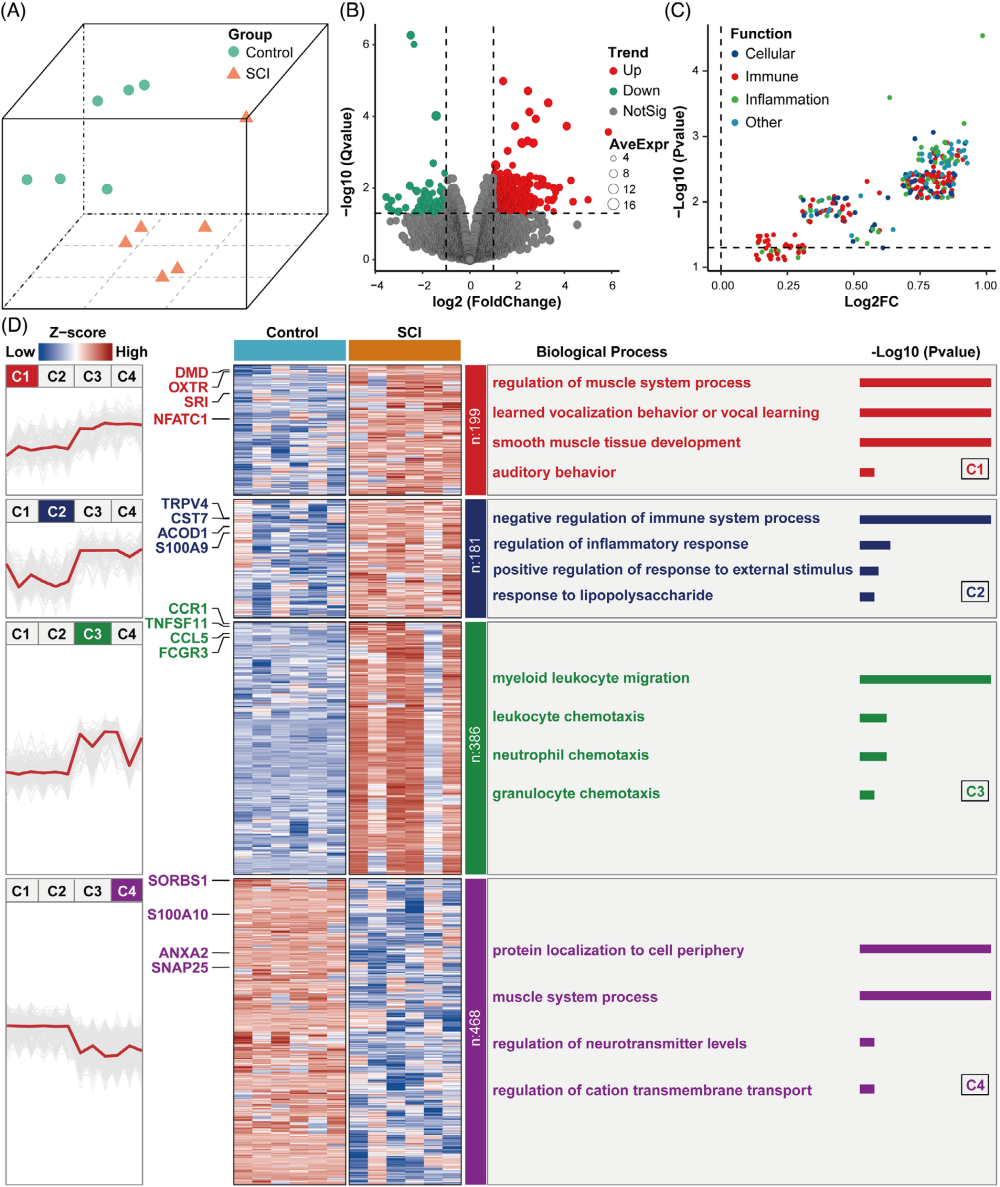
Differential gene expression and functional enrichment analysis. (A) Uniform manifold approximation and projection (UMAP) analysis plot of normalized gene chip GSE5296. (B) Volcano plot of the results of the differential gene expression analysis. (C) Volcano plot of differential expression of gene set variation analysis (GSVA); colors represent different biological process. (D) Heatmap of trend clustering results; the left part shows the trend clustering results; the middle part shows the heatmap of the expression of the genes in the four clusters (C1, C2, C3 and C4); and the right part is divided into three columns from left to right: the first column is the number of genes included in each cluster (C1, C2, C3 and C4); the second column is the biological process of each cluster; and the third column is the −log10 *p* value of the corresponding biological process term.

To analyse the overall gene expression profile of mice with acute SCI, we comprehensively analysed RNA‐sequencing data from the spinal cords of subjects in the control and SCI groups in the GSE5296 dataset, using background correction. The UMAP results showed that in the normalised microarray data, the control and SCI groups have excellent segregation, which indicates that the normalisation is successful (Figure 1A). To evaluate the molecular mechanisms of the changes induced by acute SCI, DEGs among the control and SCI groups were identified. We found that 766 genes were significantly up‐regulated and 468 genes were significantly down‐regulated after SCI based on cutoffs of a *Q*‐value of < .05 and an absolute log fold change ≥ 1.0 (Figure 1B). Next, we analysed the effects of DEGs on biologically relevant functions using GSVA analysis. The GSVA results of DEGs revealed that biological processes were significantly up‐regulated (adjusted *P*‐value < .05), including cellular, immune, inflammation and other biologically relevant functions (Figure 1C). We defined four SCI subtypes named C1, C2, C3 and C4 subsequently, and the numbers of DEGs contained in these four subtypes were 199 (C1), 181 (C2), 386 (C3) and 468 (C4), respectively. The results showed that the expression trends in the C1, C2 and C3 clusters were up‐regulated, whereas the expression trend in the C4 cluster was down‐regulated. (Figure 1D). The GSVA results indicated that biological processes such as immunity and inflammation were significantly up‐regulated after SCI. Naturally, the up‐regulated subtypes are our focus, namely C1, C2,, and C3. Furthermore, we performed functional enrichment analysis in each of the four clusters. The results indicated that biological processes such as: regulation of the muscle system process and learned vocalisation behaviour or vocal learning were significantly enriched in the C1 cluster; negative regulation of the immune system process and regulation of inflammatory response were significantly enriched in the C2 cluster, myeloid leukocyte migration and leukocyte chemotaxis enriched in the C3 cluster, and protein localisation to cell periphery and muscle system process enriched in the C4 cluster (Figure 1D). Based on the results of GSVA, the C2 cluster played an important role in immune and inflammation‐related biological functions, and the genes ACOD1, TRPV4, CST7 and S100A9 may dominate inflammatory and immune‐related functions after SCI.

### 3.2 Neural debris and spinal cord injury induce increased expression of ACOD1 and Nrf2 during neuroinflammation

**FIGURE 2:**
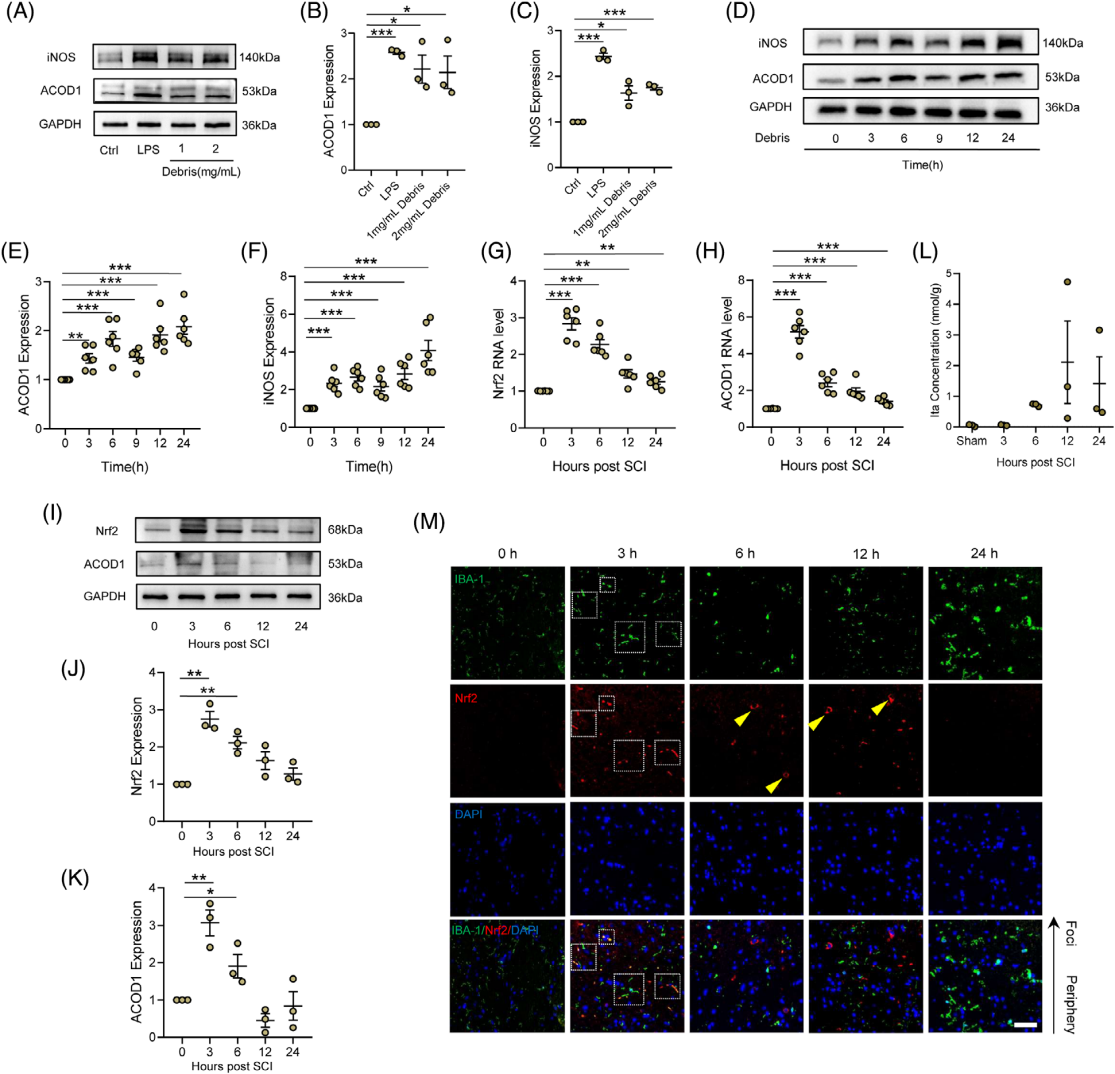
Neural debris and SCI induce increased expression of ACOD1 and Nrf2 during neuroinflammation. (A) Western blotting of iNOS and ACOD1 expression in microglia treated with LPS (1 μg/mL) and debris (1 mg/mL or 2 mg/mL) for 24 h. (B,C) Densitometric analysis of iNOS and ACOD1 expression. (D) Western blotting of iNOS and ACOD1 expression in microglia treated with debris (2 mg/mL) within 24 h. (E,F) Densitometric analysis of iNOS and ACOD1 expression. (G) Relative mRNA level of Nrf2 in the spinal cord within 24 h post‐SCI. (H) Relative mRNA level of ACOD1 in the spinal cord within 24 h post‐SCI. (I) Western blotting of Nrf2 and ACOD1 protein levels in the spinal cord within 24 h post‐SCI. (J,K) Densitometric analysis of Nrf2 and ACOD1 expressions. (L) The level of Ita in the spinal cord within 24 h post‐SCI. (M) Representative immunofluorescence labelling images of IBA‐1 (green) and Nrf2 (red) in the spinal cord within 24 h post‐SCI; scale bar = 50 μm. Data are representative of at least three biological replicates. Data are shown as mean ± SEM, and statistical significance was determined with one‐way ANOVA followed by the Tukey's post hoc test. *, *p* < .05, **, *p* < .01, ***, *p* < .001. SCI, Spinal cord injury; ACOD1, Aconitate decarboxylase 1; LPS, lipopolysaccharide.

LPS has been used to induce both an inflammatory environment and microglial activation.^36^, ^37^ Here, to explore a more appropriate stimulant, we employed neural debris isolated from spinal cord tissue to induce microglia activation in vitro. The results showed that 1 and 2 mg/mL of neural debris provoked a significant increase in the expression of inducible nitric oxide synthase (iNOS)—a biomarker of inflammatory microglia—and ACOD1 (Figure 2A,C). Although the debris‐mediated increase in the iNOS expression in microglia was weaker than treatment with 1 μg/mL LPS, the expression of ACOD1 in each treatment group showed little difference (Figure 2A,B). Furthermore, after microglia were treated with 2 mg/mL of neural debris, the expression of iNOS and ACOD1 was determined by the western blot. At 3 h post debris treatment, the expression of iNOS and ACOD1 significantly increased, a trend that continued until 24 h, although there was an inconspicuous decrease at 9 h (Figure 2D–F). PCR results showed that Nrf2 and ACOD1 mRNA expression peaked at 3 h and gradually decreased in the SCI model of mouse within 24 h (Figure 2G,H). However, Nrf2 and ACOD1 protein expression showed a decreasing trend within 24 h post stimuli, but which showed consistent and significant increases at 3 and 6 h post‐SCI (Figure 2I–K). Unexpectedly, the level of Ita did not increase at 3 h and began to elevate at 6 h post‐SCI (Figure 2L). Next, to better understand Nrf2 expression in microglia, we performed double‐immuno‐labelling of Nrf2 and the microglial marker IBA‐1 at 24 h post‐SCI. The results indicated that the increased Nrf2 expression in microglia (marked using the white dotted frames) was apparent at 3 h post‐SCI, but at other timings we seldom observed Nrf2/IBA‐1 double‐stained cells (Figure 2M). Interestingly, the cells that were positive for Nrf2 presented with a neuron‐like shape (marked using yellow arrows) at 6 and 12 h post‐SCI (Figure 2M), indicating that the expression of Nrf2 located principally in neurons from 6 to 12 h post‐SCI. Taken together, the results suggest that ACOD1, rather than Ita, induces an increase of the Nrf2 expression in microglia under inflammatory conditions post SCI.

### 3.3 AOCD1 silencing results in cumulative dysfunction of the redox reaction in microglia

**FIGURE 3:**
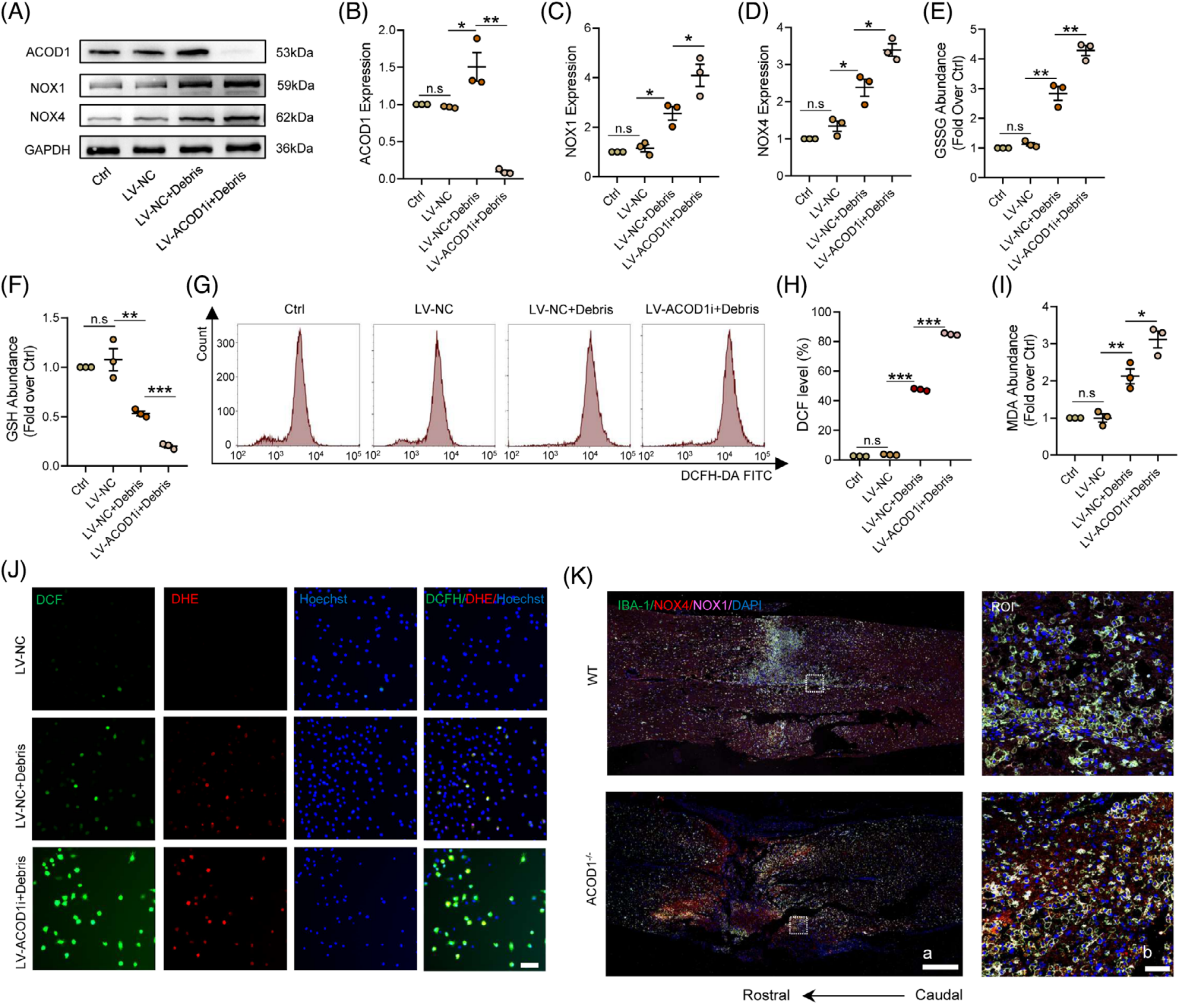
ACOD1 loss results in cumulative dysfunction of the redox reaction in microglia. (A) Western blotting of ACOD1, NOX1 and NOX4 expression in microglia treated with debris (2 mg/mL) for 24 h after transfection with ACOD1i. (B–D) Densitometric analysis of ACOD1, NOX1 and NOX4 expression. (E) Relative GSSG levels in microglia treated with debris (2 mg/mL) for 24 h after transfection with ACOD1i. (F) Relative GSH levels in microglia treated with debris (2 mg/mL) for 24 h after transfection with ACOD1i. (G) Flow cytometry and analysis of ROS levels in microglia. (H) Quantitative analysis of ROS expression. (I) Relative MDA content in microglia treated with debris (2 mg/mL) for 24 h after transfection with ACOD1i. (J) Representative immunofluorescence labelling images of DCF (green) and DHE (red) in microglia treated with debris (2 mg/mL) for 24 h after transfection with ACOD1i; Scale bar = 70 μm. (K) Representative immunofluorescence labelling images of IBA‐1 (green), NOX4 (red) and NOX1 (pink) obtained from longitudinal sections centred around the injured core 5 mm in WT and ACOD1^−/−^ mice at 3 days post‐SCI; scale bar *a* = 500 μm, *b* = 50 μm. Data are representative of at least three biological replicates. Data are shown as mean ± SEM, and statistical significance was determined with one‐way ANOVA followed by Tukey's post hoc test. *, *p* < .05, **, *p* < .01, ***, *p* < .001, n.s, No significance; ROI, region of interest; SCI, spinal cord injury; ACOD1, aconitate decarboxylase 1; ROS, reactive oxygen species.

To comprehensively investigate the multifunctional effects of AOCD1 in microglia during neuroinflammation, we utilised shRNA‐ACOD1 and cDNA‐ACOD1 to achieve AOCD1 interference (ACOD1i) and ACOD1 overexpression, respectively, prior to the neural debris treatment. The results showed that transfection of ACOD1 cDNA did not increase ACOD1 protein levels, but that ACOD1i significantly decreased ACOD1 expression, after the debris treatment (Additional file 1: Figure S1A,B). We first examined OS levels in debris‐treated microglia after ACOD1i and found that the NADPH oxidases NOX1 and NOX4 increased after the debris treatment, but that ACOD1i caused NOX1 and NOX4 expression markedly higher (Figure 3A,C,D). Moreover, the level of the redox reaction, as reflected by oxidised glutathione (GSSG)/glutathione (GSH) content, indicated that the debris treatment contributed to a prominent increase in the GSSG content and decrease in that of GSH. Importantly, ACOD1i significantly increased the GSSG content and decreased that of GSH in microglia (Figure 3E,F). Flow cytometry results indicated that, after the neural debris treatment, ROS levels increased to approximately 50% of baseline levels in microglia and increased to over 80% in ACOD1i‐pretreated microglia (Figure 3G,H), suggesting that ACOD1i aggravated dysfunction of mitochondria after the debris treatment. Besides, ELISA results indicated that the MDA content significantly increased in ACOD1i‐pretreated microglia companied to the NC microglia after the debris treatment (Figure 3I). Furthermore, dichlorofluorescein (DCF)/dihydroethium (DHE)‐double staining showed that the debris treatment resulted in a part of ROS positive microglia in the LV‐NC group and a more obvious increase in the ACOD1i group (Figure 3J). In vivo, the mRNAs levels of NOX1, NOX4 and HO‐1 were examined by PCR at 3 h post‐SCI and results indicated that ACOD1i prominently increased the mRNA expression of NOX1 and NOX4 and decreased that of the downstream gene HO‐1, which is regulated by Nrf2 (Additional file 1: Figure S2A–C). At 3 days post‐SCI, microglia in WT mice, which had accumulated around the injury foci, expressed NOX1 and NOX4; however, microglia in *ACOD1^−/−^* mice had significantly increased NOX1 and NOX4 expression levels around the injury foci compared with their WT counterparts (Figure 3K). In summary, the results indicated that AOCD1i enhances OS‐induced cellular dysfunction in microglia during neuroinflammation both in vitro and in vivo.

### 3.4 ACOD1i increases neuroinflammation and acceleration of glial‐cell accumulation

**FIGURE 4:**
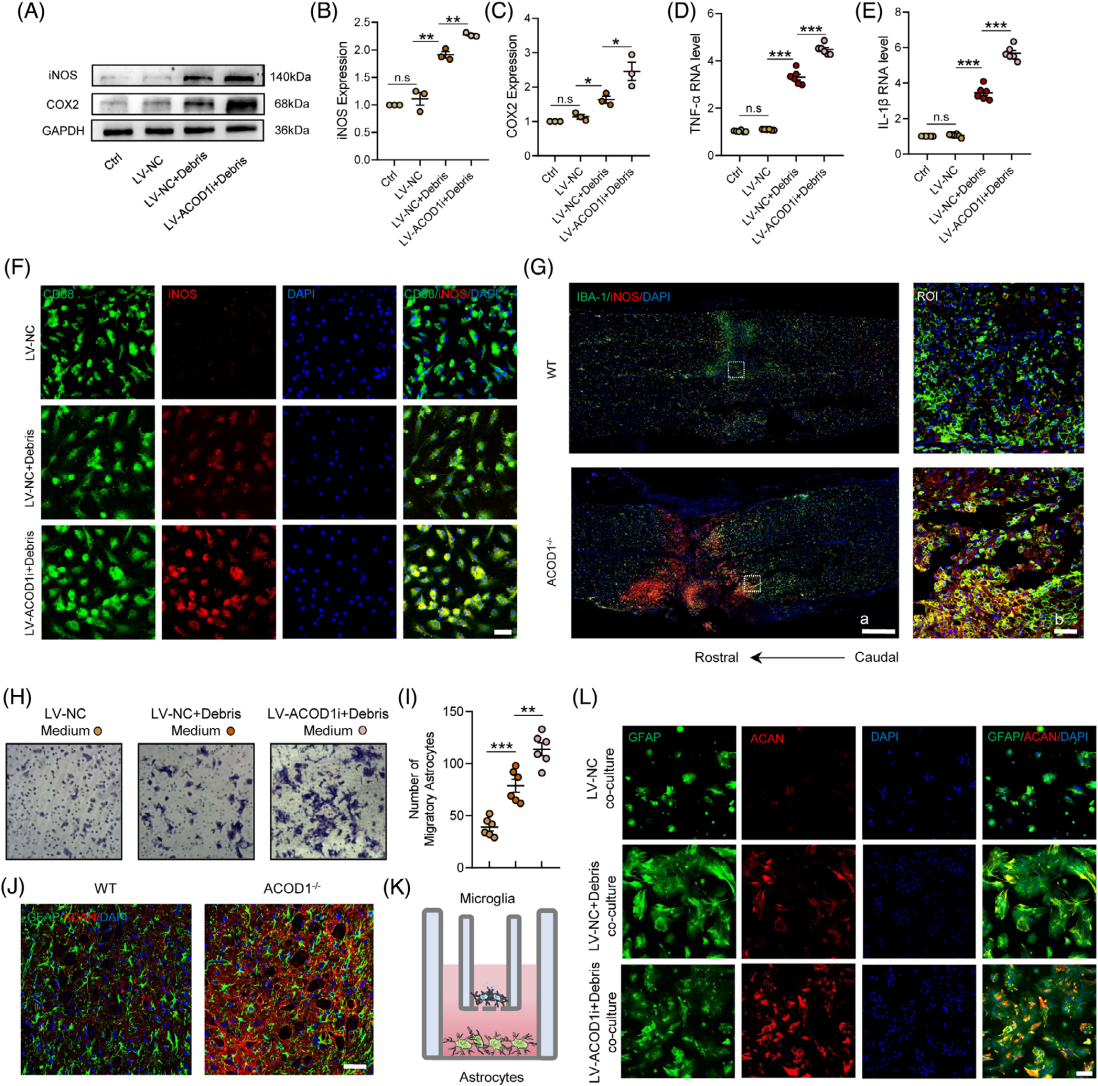
ACOD1 loss increases neuroinflammation and acceleration of glial accumulation. (A) Western blotting of iNOS and COX2 in microglia treated with debris (2 mg/mL) for 24 h after transfection with ACOD1i. (B,C) Densitometric analysis of iNOS and COX‐2 expression. (D,E) Relative mRNA levels of TNF‐α and IL‐1β in microglia treated with debris (2 mg/mL) for 24 h after transfection with ACOD1i. (F) Representative immunofluorescence labelling images of CD68 (green) and iNOS (red) in microglia treated with debris (2 mg/mL) for 24 h after transfection with ACOD1i; scale bar = 30 μm. (G) Representative immunofluorescence labelling images of IBA‐1 (green) and iNOS (red) obtained from longitudinal sections centred around the injured core 5 mm in WT and ACOD1^−/−^ mice at 3 days post‐SCI; scale bar *a* = 500 μm, *b* = 50 μm. (H) Representative images of astrocytes stained with crystal violet in trans‐well assay, which were treated with microglial medium for 72 h. (I) Quantitative analysis of the amounts of astrocytes under inserts. (J) Representative immunofluorescence labelling images for GFAP (green) and ACAN (red) obtained from longitudinal sections centred around the injured core 5 mm in WT and ACOD1^−/−^ mice at 7 days post‐SCI; scale bar = 50 μm. (K) Coculture of primary microglia and astrocytes. (L) Representative immunofluorescence labelling images of GFAP (green) and ACAN (red) in astrocytes co‐cultured with debris‐stimulated microglia for 24 h after transfection with ACOD1i; scale bar = 100 μm. Data are representative of at least three biological replicates. Data are shown as mean ± SEM, and statistical significance was determined with one‐way ANOVA followed by Tukey's post hoc test. *, *p* < .05, **, *p* < .01, ***, *p* < .001, ns, No significance; ROI, region of interest; SCI, spinal cord injury; ACOD1, aconitate decarboxylase 1; WT, wild‐type; ACAN, aggrecan.

Previous research has demonstrated that AOCD1 plays a potent anti‐inflammatory role in human macrophages in vitro. Considering that microglia are the resident macrophages in the nervous system, we tested whether silencing AOCD1 can alter the response of microglial to an inflammatory environment. Indeed, ACOD1i caused increased expression of iNOS and cyclooxygenase 2 (COX‐2) (two classic inflammatory mediators) during microglial activation (Figure 4A–C). As expected, after the debris treatment, the mRNA expression of TNF‐α, IL‐1β and IL‐6 due also increased (Figure 4D,E; Additional file 1: Figure 1C), suggesting that microglia lacking ACOD1 had a stronger inflammatory response after the debris treatment in vitro. Of note, at 24 h after the debris treatment, IF staining results showed the increased iNOS expression in microglia (Figure 4F). Similar to the above in vitro results, *ACOD1^−/−^* mice had higher microglial‐derived iNOS expression and a more extensive inflammatory area at 3 days post‐SCI, compared with WT mice (Figure 4G). Moreover, changes in inflammatory cytokines including TNF‐α, IL‐1β and IL‐6 were markedly increased in *ACOD1^−/−^* mice (Additional file 1: Figure S2D–F). We next analysed how ACOD1 deficiency affects glial scarring. Specifically, we employed a microglial inflammation model to analyse reactive astrocytes migration and related metabolites such as aggrecan (ACAN). The astrocytes cultured in the medium from ACOD1i microglia had migrated in greater numbers and travelled longer distances than astrocytes cultured in the control medium (Figure 4H,I). Coculture results, shown in Figure 4K, indicated that astrocytes cocultured with ACOD1i microglia had stronger expression of ACAN than those cocultured with control microglia after the debris treatment (Figure 4L). Likewise, astrocytes secreted more ACAN in *ACOD1^−/−^* mice at 7 days post‐SCI (Figure 4J).

### 3.5 Axonal and locomotor dysfunction increases in ACOD1^−/−^ mice post‐SCI

**FIGURE 5:**
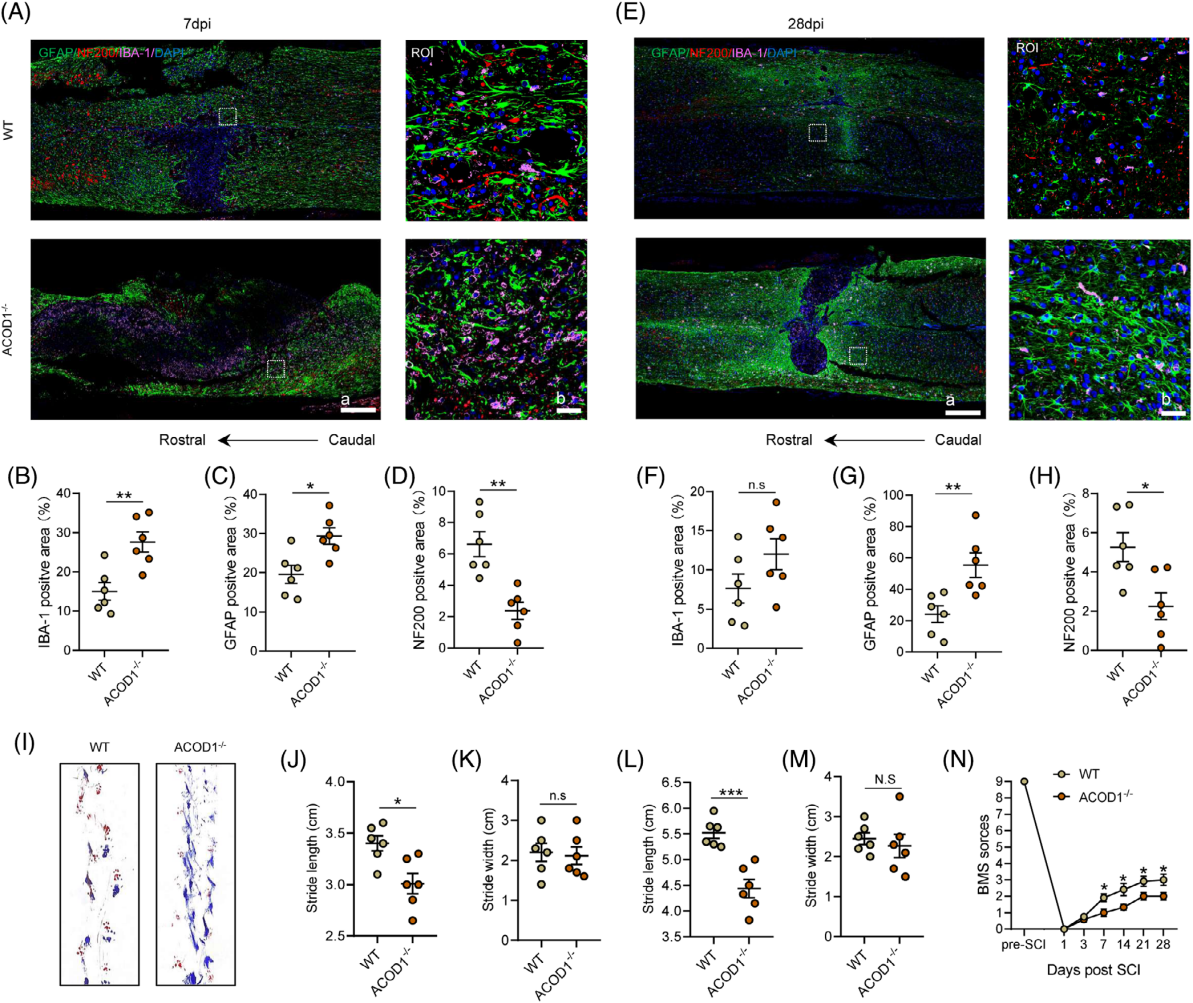
Histological collapse and locomotor dysfunction become worse in ACOD1^−/−^ mice after SCI. (A) Representative immunofluorescence labelling images of GFAP (green), NF200 (red) and IBA‐1 (pink) obtained from longitudinal sections centred around the injured core 5 mm in WT and ACOD1^−/−^ mice at 7 days post‐SCI; scale bar *a* = 500 μm, *b* = 50 μm. (B) Quantitative analysis of the area of microglial scar at 7 days post‐SCI. (C) Quantitative analysis of the astroglial scar at 7 days post SCI. (D) Quantitative analysis of the axonal numbers at 7 days post‐SCI. (E) Representative immunofluorescence labelling images of GFAP (green), NF200 (red) and IBA‐1 (pink) obtained from longitudinal sections centred around the injured core 5 mm in WT and ACOD1^−/−^ mice at 28 days post SCI; scale bar *a* = 500 μm, *b* = 50 μm. (F) Quantitative analysis of the area of the microglial scar at 28 days post‐SCI. (G) Quantitative analysis of the area of the astroglial scar at 28 days post‐SCI. (H) Quantitative analysis of the axon area at 28 dpi. (I) A footprint analysis of WT and ACOD1^−/−^ mice performed at 7 days post‐SCI. (J,M) Quantification of the footprint analysis at 7 and 28 days post SCI in WT and ACOD1^−/−^ mice. (N) The BMS score within 28 days post‐SCI in WT and ACOD1^−/−^ mice. Data are representative of at least three biological replicates. Data are shown as mean ± SEM, and statistical significance was determined with one‐way ANOVA followed by Tukey's post hoc test. *, *p* < .05, **, *p* < .01, ***, *p* < .001, ns, no significance; ROI, region of interest; SCI, spinal cord injury; ACOD1, aconitate decarboxylase 1; WT, wild‐type.

Given the important role of microglia in neurological remodelling post‐SCI, we examined glia scar formation and axonal growth at 7 and 28 days post‐SCI. At 7 days post‐SCI, compared with the WT mice, a larger secondary injury area (Additional file 1: Figure S3A,B) and increased number of microglia/macrophages around the injury foci were apparent in *ACOD1^−/−^* mice (Figure 5A,B), along with a more dense glia scar formation (Figure 5A,C). Likewise, fewer axons were observed surrounding the injury epicenter in *ACOD1^−/−^* mice compared with their WT counterparts (Figure 5A,D). At 28 days post‐SCI, no significant difference was found in the microglia positive area between the two groups, but a significant increase in glial scar formation and marked decrease in the axonal network were found *ACOD1^−/−^* mice, along with a lager collapse of neurohistology (Figure 5E–H; Additional file 1: Figure S3A,C). Besides, *ACOD1^−/−^* mice had significantly reduced myelin sheaths than WT mice both at 7 and 28 days post‐SCI (Additional file 1: Figure S3D–F), but there was no significant difference in neuronal survival within a 500 μm radius of the injured foci between the two groups (Additional file 1: Figure S3G–I). We next analysed the results of the footprint and BMS assays to assess locomotor differences. As shown in Figure 5I, at 7 days post‐SCI, compared with WT mice, *ACOD1^−/−^* mice had a more frequent drag of the instep and shorter stride length, although the difference in stride width was not significant (Figure 5J,K). Likewise, the data for stride length and width at 28 days post‐SCI were similar to that at 7 days post‐SCI (Figure 5L,M). Results of the BMS assay indicated no significant difference in the scores between the two groups at 3 days post‐SCI; however, from 7 to 28 days post‐SCI, the scores of the *ACOD1^−/−^* mice were significantly lower than those of WT mice (Figure 5N).

### 3.6 ACOD1 promotes Keap1/Nrf2 complex disruption by activating p62 phosphorylation at Ser351

**FIGURE 6:**
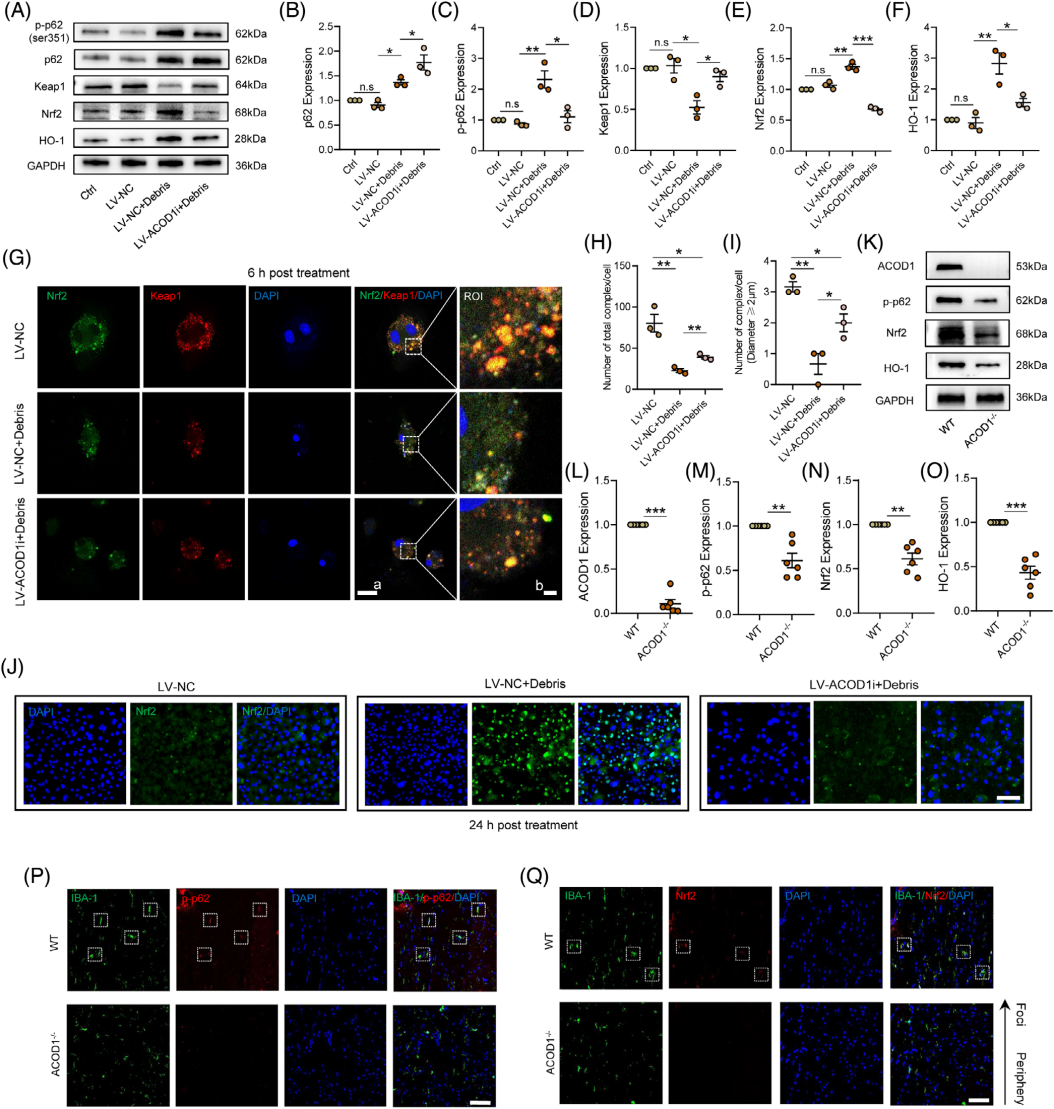
ACOD1 promotes Keap1/Nrf2 complex disruption by activating p62 phosphorylation at Ser351. (A) Western blotting of p‐p62, p62, Keap1, Nrf2 and HO‐1 expression in microglia treated with debris (2 mg/mL) for 24 h after transfection with ACOD1i. (B–F) Densitometric analysis of p‐p62, p62, Keap1, Nrf2 and HO‐1 expression. (G) The colocalisation of Nrf2 and Keap1 in microglia treated with debris (2 mg/mL) for 24 h after transfection with ACOD1i was evaluated by immunofluorescence; scale bar *a* = 20 μm, *b* = 2 μm. (H,I) Quantitative analysis of Keap1–Nrf2 complex number. (J) The colocalisation of Nrf2 and DAPI in microglia treated with debris (2 mg/mL) for 24 h after transfection with ACOD1i was evaluated by immunofluorescence; scale bar *a* = 20 μm, *b* = 2 μm. (K) Western blotting of ACOD1, p‐p62, Nrf2 and HO‐1 expression in WT and ACOD1^−/−^ mice at 3 h post‐SCI. (L–O) Densitometric analysis of ACOD1, p‐p62, Nrf2 and HO‐1 expression. (P) Representative immunofluorescence labelling images of IBA‐1 (green) and p‐p62 (red) in the spinal cords of WT and ACOD1^−/−^ mice at 3 h post‐SCI; scale bar = 50 μm. (Q) Representative immunofluorescence labelling images of IBA‐1 (green) and Nrf2 (red) in the spinal cords of WT and ACOD1^−/−^ mice at 3 h post‐SCI; scale bar = 50 μm. Data are representative of at least three biological replicates. Data are shown as mean ± SEM, and statistical significance was determined with one‐way ANOVA followed by Tukey's post hoc test. *, *p* < .05, **, *p* < .01, ***, *p* < .001, ns, no significance; ROI, region of interest; SCI, spinal cord injury; ACOD1, aconitate decarboxylase 1; WT, wild‐type.

ACOD1 and its metabolite have been identified as an alkylation promoter of Keap1, contributing to an anti‐inflammatory role in macrophages. Considering that there are various signalling molecules that regulated the Keap1/Nrf2 complex, we hypothesised whether ACOD1 regulates Nrf2 though its upstream molecule p62. As expected, ACOD1i significantly decreased the phosphorylation of p62 and increased p62 protein levels (Figure 6A–C). After the debris treatment, the decreased expression of Nrf2 and its classic downstream factor HO‐1 due to ACOD1i were consistent with the reduced level of p‐p62, along with an opposite alteration of Keap1 protein (Figure 6A,D–F). The IF result showed that ACOD1i caused an obvious dedimerisation between p‐p62 and Keap1 in debris‐insulted microglia (Additional file 1: Figure S4A). Furthermore, confocal microscopy was utilised to distinguish the distribution of the Keap1/Nrf2 complex in the cytoplasm following debris treatment‐induced microglial activation, with results indicating that activation promoted the Keap1/Nrf2 complex dysfunction but that ACOD1i reversed the process at the 6 h post debris treatment (Figure 6G–I). Moreover, we found that ACOD1i prominently inhibited Nrf2 nuclear translocation at the 24 h post debris treatment (Figure 6J). In vivo, *ACOD1^−/−^* mice also displayed a marked decrease in phosphorylation of p62, as well as in the expression of p62, Nrf2 and HO‐1 at 3 days post‐SCI compared with WT mice (Figure 6K, M–O). Likewise, IF staining results indicated reduced the expression of p‐p62 and Nrf2 in microglia isolated from *ACOD1^−/−^* mice at 3 days post‐SCI (Figure 6P,Q). Taken together, loss of ACOD1 causes a decrease of the p‐p62 expression in microglia post‐SCI, resulting in a prominent inhibition of Nrf2 activation.

### 3.7 A positive feedback loop of ACOD1/p‐p62/Nrf2/ACOD1 was identified in microglia during neuroinflammation

**FIGURE 7:**
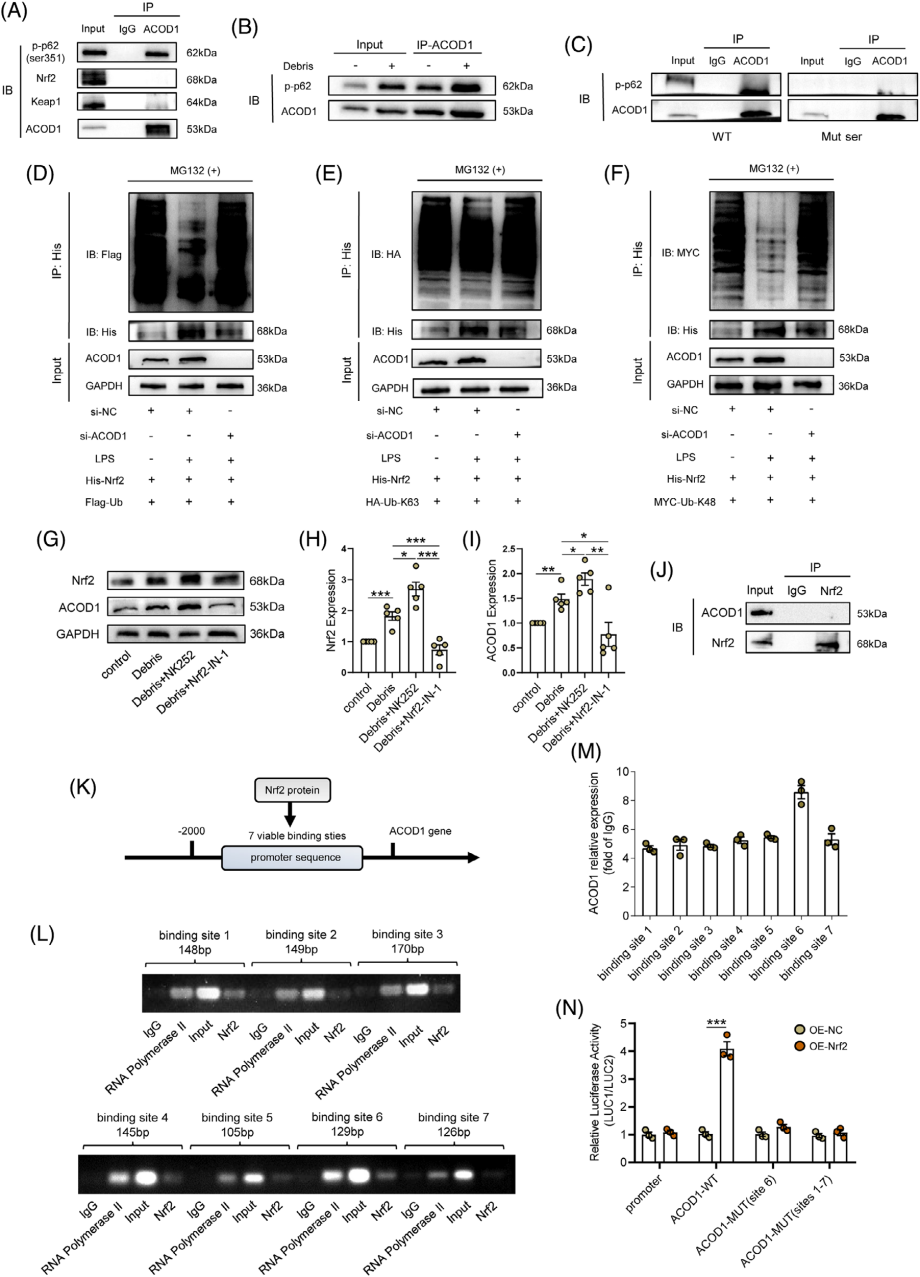
A positive feedback loop of ACOD1/p‐p62/Nrf2/ACOD1 was identified in microglia during neuroinflammation. (A) Co‐IP assay showing representative protein bands of p‐p62, Nrf2, Keap1 and ACOD1 in microglia after using an Ab against ACOD1. (B) Co‐IP assay showing representative protein bands of p‐p62 and ACOD1 in microglia treated with debris (2 mg/mL) or not using an Ab against ACOD1. (C) Co‐IP assay showing representative protein bands of p‐p62 and ACOD1 in WT or Mut ser using an Ab against ACOD1. (D) Representative protein bands of ubiquitin bound to Nrf2 using the Co‐IP assay. (E) Representative protein bands of ubiquitin linkage‐specific K63 bound to Nrf2 using the Co‐IP assay. (F) Representative protein bands of ubiquitin linkage‐specific K48 bound to Nrf2 using the Co‐IP assay. (G) Western blotting of Nrf2 and ACOD1 in microglia pretreated with NK252 or Nrf2‐IN‐1 and then treated with debris (2 mg/mL) for 24 h. (H,I) Densitometric analysis of Nrf2 and ACOD1 expression. (J) Co‐IP assay showing representative protein bands of Nrf2 and ACOD1 in microglia. (K) Seven binding sites between Nrf2 and the ACOD1 promoter using the Jaspar website. (l) ChIP assay shows binding at predicted binding sites between Nrf2 and ACOD1 promoters. (M) Densitometric analysis of ACOD1 expression. (N) The luciferase activity of ACOD1 treated with Nrf2 overexpression or not after mutation in the 6th promoter site 1st–7th promoter sites. Data are shown as mean ± SEM, and statistical significance was determined with one‐way ANOVA followed by Tukey's post hoc test. *, *p* < .05, **, *p* < .01, ***, *p* < .001. ACOD1, aconitate decarboxylase 1; WT, wild‐type; ChIP, chromatin immunoprecipitation.

We explored the binding interaction between ACOD1 and proteins that are involved in the Nrf2 activation pathway, and found that ACOD1 interacted with p‐p62 rather than Nrf2 or Keap1, an interaction that was enhanced in microglia after the debris treatment (Figure 7A,B). The results suggested that ACOD1 regulates the Nrf2 activation by binding directly to p‐p62. Furthermore, we transfected a mutant of p62 gene in microglia, in which the encoding gene of its 351th serine was replaced by GCT (Additional file 1: Table S2). The IP results showed vanish not only in the expression of p‐p62 but in the binding between ACOD1 and p‐p62 (Figure 7C). To identify the role of ACOD1 in Nrf2 ubiquitination, we transfected HEK‐293T cells with a His‐tagged Nrf2 overexpression vector, Flag‐tagged ubiquitin overexpression vector, MYC‐tagged ubiquitin linkage‐specific K48 overexpression vector, and HA‐tagged ubiquitin linkage‐specific K63 overexpression vector line prior to the LPS treatment, and the degree of Nrf2 ubiquitination was then determined after ACOD1i. As shown in Figure 7D, the LPS treatment increased the His‐Nrf2 expression, decreased Flag‐ubiquitin, and increased the ACOD1 expression; however, the expressions of His‐Nrf2 and Flag‐ubiquitin were reversed after ACOD1i. Interestingly, there was a consistent change in MYC‐labelled ubiquitin linkage‐specific K48 in each group, but little change was evident in HA‐ ubiquitin linkage‐specific K63 (Figure 7E,F), suggesting that ACOD1 affected ubiquitination of Nrf2 via ubiquitin linkage‐specific K48. Given the role of ACOD1 in p‐p62‐induced Nrf2 activation, we further examined the effects of Ita and 4‐OI on p62 expression and its phosphorylation as well as LC3II/I expression in microglia treated with debris after ACOD1i. Ita or 4‐OI supplement strongly reduced the phosphorylation of p62 at Ser351 in microglia without ACOD1i; they seldom changed the expression of p‐p62 when ACOD1 was silenced, but silencing ACOD1 reduced the p‐p62 level at Ser351 in either Ita‐ or 4‐OI‐treated microglia (Additional file 1: Figure S4E,F). Interestingly, the expression of p62 was similar in ACOD1i microglia, but the expression of p62 was reduced in either Ita‐ or 4‐OI‐treated ACOD1i microglia (Additional file 1: Figure S4E,G). Besides, an increased expression in nuclear Nrf2 protein and decreased expression in cytoplasmic Nrf2 was evident, which were reversed in ACOD1i microglia (Additional file 1: Figure S4B–D). Next, we examined the role of Nrf2 in the ACOD1 expression using the Nrf2 activator NK252 and Nrf2 inhibitor Nrf2‐IN‐1, which as expected, increased and decreased the expression of Nrf2, respectively. Interestingly, the expression of ACOD1 followed a similar pattern after the debris treatment (Figure 7G–I). However, the IP assay showed that Nrf2 protein did not directly interact with ACOD1 (Figure 7J). We therefore speculated that Nrf2, as a transcription factor, affected the ACOD1 expression by regulating its mRNA transcription. Furthermore, we predicted seven binding sites between Nrf2 and the ACOD1 promoter using the Jaspar website (Figure 7K). Next, the potential ACOD1 promoter binding sites were verified by a ChIP assay, and the 6th promoter site of the ACOD1 gene that bound Nrf2 showed a binding efficiency two time that of the sites (Figure 7L,M). To further confirm the link between Nrf2 and the 6th promoter site of the ACOD1 gene, a luciferase report assay was carried out mutation of the promotor binding sites in a BV_2_ microglia line. The result showed that the luciferase activity of ACOD1 significantly increased after the Nrf2 overexpression; however, such increase in ACOD1 was negated after mutation in the 6th promoter site 1st –7th promoter sites (Figure 7N). Overall, the results indicated that Nrf2 promotes the transcription of ACOD1 via binding with the 6th promoter site.

## 4 DISCUSSION

Currently, methylprednisolone administration, within 6 h after SCI, is the only ant‐inflammatory treatment available in clinic^38^, ^39^, ^40^; however, the treatment has not only failed to significantly improve neuropathology, but has also caused adverse reactions, such increased infection and hyperglycemia.^40^, ^41^, ^42^ As a consequence, endogenous targets against neuroinflammation have been studied to help develop therapies for SCI. Herein, we identified ACOD1 as an early neuroprotective marker after SCI, which has anti‐inflammatory/oxidative capabilities in microglia‐triggered neuroinflammation. Mechanistically, ACOD1 regulates Keap1 degradation and Nrf2 activation by binding with phosphorylated p62 at Ser351, after which Nrf2 further increases the transcription of ACOD1, forming a feedback loop.

Multiple studies that concern post‐SCI neuroinflammation has showed a classic inflammatory stimulation of microglia by LPS in vitro,^43^, ^44^, ^45^ but translational ability of such treatment has been questioned in regard to SCI‐mediated neuroinflammation. Considering that LPS is a product derived from the outer cytoderm of Gram‐negative bacteria, it functions as a pathogen‐associated molecular pattern to elicit innate immunity.^46^, ^47^ However, SCI‐induced secondary neuroinflammation is initiated by destroyed tissue and cellular components.^48^ In the present study, to distinguish from other central nervous system disorders caused by pathogenic infection, we‐used neural debris from spinal cord tissue to mimic SCI‐mediated aseptic inflammation in vitro. Increased ACOD1 expression has been previously reported to be involved in the initiation and activation of microglia^49^; however, ACOD1 expression patterns change in inflammatory microglia has not been studied. We found that treatment with cord debris resulted in microglia expressing a similar level of the ACOD1 expression and inflammatory level, when compared with those treated with LPS. Besides, the debris treatment caused a persistently high level of iNOS and ACOD1 expression in microglia from 3 to 24 h after stimulation. The results suggest that ACOD1 may be associated with the development of inflammation in debris‐treated microglia. In mouse RAW264.7 macrophages, ACOD1 transcription occurs as early as 1.5 h after LPS insult, and the peaks at 4–6 h after stimulation.^14^, ^50^ Although, this expression timeframe is similar to both the transcription and the translation of ACOD1 and Nrf2 our SCI mice, it was different from the ACOD1 protein expression profile in microglia treated with debris. The results indicated that there are several differences between macrophage lines and primary microglia in regard to the ACOD1 expression during inflammation, and that the complex interactions between multiple cells in vivo may account for the difference in the ACOD1 expression between the in vitro and in vivo models.

Following SCI, activated microglia undergo a metabolic reprogramming by increasing glycolysis instead of oxidative phosphorylation in response to primary injury, which further produces ROS for the host defense.^51^, ^52^ Oxidative damage by ROS kills invading pathogens and induces increased expression of genes involved in inflammatory modulation.^53^, ^54^ Interestingly, a study showed that ACOD1 regulates the ROS generation and promotes A20 expression, which inhibits NF‐κB signals to limit release of inflammatory cytokines.^18^, ^55^ However, in contrast, activation of STAT1/3 by ACOD1‐induced ROS also caused cytokine accumulation.^56^ Inhibition of ACOD1 causes microglia forming a protective subtype and promotes neuronal regeneration after parasite or virus infection.^24^, ^57^ Although the pathogenesis of post‐SCI neuroinflammation is triggered by mechanical injury‐induced fragmentised cells and tissue, our findings also suggested that the absence of ACOD1 aggravated OS and the inflammatory response in either SCI mice or debris‐treated microglia. Besides, a recent study suggested that ACOD1/Ita reduces microglia‐induced inflammation in SCI mice,^26^ but it did no such evidence regarding ROS‐induced OS has been found.

Although ACOD1/Ita exerts an anti‐inflammatory effect through activation of Nrf2 in various disease models including bone injury,^58^, ^59^ liver injury^23^, ^60^ and neurological disorders,^26^, ^60^ the molecular mechanism is based on the promotion of alkylation of Keap1 in macrophages.^19^ However, the molecular pathway whereby ACOD1 regulates the Nrf2 expression in activated microglia during neuroinflammation is not fully elucidated. SQSTM1/p62 is a substrate protein of autophagy, and although p62 directly interacts with the 349‐DPSTGE‐354 motif in the Keap1‐interaction region, its phosphorylation also limits the protein interaction of the Keap1–Nrf2 complex.^61^, ^62^ Overall, this complex process further enhances Nrf2 deubiquitylation leading to activation of Nrf2‐mediated anti‐oxidant/inflammatory signals.^63^ Our data showed that ACOD1 could directly interact with phosphorylated p62 at Ser351 to promote p62‐induced autophagy to degrade Keap1, which further impaired Nrf2 ubiquitination by Keap1 to retain increased levels of both nuclear and cytoplasmic Nrf2. Unexpectedly, treatment with Ita or 4‐OI significantly reduced the level of phosphorylated p62 and inhibited the development of autophagy, indicating that p‐p62‐induced Keap1 degradation is dependent on the ACOD1 expression rather than exogenous Ita and 4‐OI. Furthermore, Ita or 4‐OI treatment did not reverse the expression of p‐p62 at Ser351 but facilitated autophagy under ACOD1i. These results indicated that the effects of ACOD1 are partly different from Ita and its derivatives, and that the properties of exogenous Ita and its derivatives may be independent of the level of ACOD1. Of note, Nrf2, as a transcription factor, regulates transcription of various protective genes, such as NQO1, HO‐1 and SOD^64^, ^65^ however, it is not clear whether Nrf2 regulates the transcription of ACOD1. We first showed that Nrf2 bound with a sequence of the ACOD1 promoter to further enhance the ACOD1 transcription.

However, some limitations and open questions remain in our study. High ACOD1/Nrf2 expression in SCI mice (3 h post‐SCI) showed shorter time than that in vitro debris‐treated microglia (24 h post‐debris treatment), which may be attributed to the complicated microenvironment and intercellular regulatory mechanisms. Moreover, the current study focused on the mechanism of how ACOD1/Nrf2 regulates microglia‐induced neuroinflammation, but it is unclear what the role of ACOD1 and exogenous Ita are in autophagy.

Taken together, we identified a new positive feedback loop inhibiting inflammation and OS, where ACOD1 promotes p62 phosphorylation at Ser351 and further depolymerises the Keap1–Nrf2 complex. Notably, as a unique regulator, ACOD1 may positively regulate autophagy in a manner different from that of exogenous Ita. Therefore, ACOD1 is a key early target regulating neuroinflammation and OS after SCI, and future studies are needed to explain the difference between endogenous ACOD1/Ita and exogenous Ita and its derivatives.

## AUTHOR CONTRIBUTIONS

ZY.Q conceived and designed the experiments. HJ.L and L.Y funded and supervised the research. ZY.Q, MJ.X and TY.Z performed the experiments. MJ.X and TY.Z performed the data anlysis. Y.L, GS.L and YN.Z assisted the experiments. ZY.Q wrote and edited the manuscript.

## CONFLICT OF INTEREST STATEMENT

The authors declare no conflicts of interest.

## ETHICS STATEMENT

Our animal protocol was approved by the Insititutonal Animal Care and Use Committee of Jiangsu Hanjiang Biology Co.LTD in accordence with the Basel Declaration (Approval No. HJSW‐23022001).

## Supporting information


**Figure S1** (A) Western blotting for ACOD1 in microglia treated with debris (2 mg/mL) for 24 h after transfection with OE‐ACOD1 or ACOD1i. (B) The densitometry analysis of the ACOD1 expression. (C) Relative mRNA levels of IL‐6 in microglia treated with debris (2 mg/mL) for 24 h after transfection with ACOD1i.

**Figure S2** (A) Relative mRNA level of NOX1 in the spinal cord of WT and ACOD1^−/−^ mice at 3 h post‐SCI. (B) Relative mRNA level of NOX4 in the spinal cord of WT and ACOD1^−/−^ mice at 3 h post‐SCI. (C) Relative mRNA level of HO‐1 in the spinal cord of WT and ACOD1^−/−^ mice at 3 hpi. (D) Relative mRNA level of TNF‐α in the spinal cord of WT and ACOD1^−/−^ mice at 3 h post‐SCI. (E) Relative mRNA level of IL‐1β in the spinal cord of WT and ACOD1^−/−^ mice at 3 h post‐SCI. (F) Relative mRNA level of IL‐6 in the spinal cord of WT and ACOD1^−/−^ mice at 3 h post‐SCI.

**Figure S3** (A) HE staining the injured spinal cord core 3 mm obtained at 7 and 28 days post‐SCI in WT and ACOD1^−/−^ mice; scale bar = 200 μm. (B,C) Quantitative analysis of the affected area at 7 and 28 days post‐SCI. (D) LFB staining images of the injured spinal cord core 3 mm at 7 and 28 days post‐SCI in WT and ACOD1−/− mice; Scale bar = 200 μm. (E and F) Quantitative analysis of the demyelinated area at 7 and 28 days post‐SCI. (G) Nissl staining images of the injured spinal cord core 3 mm at 7 and 28 post‐SCI in WT and ACOD1^−/−^ mice; scale bar = 200 μm. (H,I) Quantitative analysis neuronal survival 7 and 28 days post‐SCI.

**Figure S4** (A) Representative IF labelling images of p‐p62 (green) and Keap1 (red) in microglia treated with debris (2 mg/mL) for 24 h after transfection with ACOD1i; scale bar = 30 μm. (B) Western blotting of Nrf2 in microglia treated with debris (2 mg/mL) for 24 h after transfection with ACOD1i. Histone was used as the control in nuclear. GAPDH was used as the control in cytosol. (C,D) Densitometric analysis of the Nrf2 expression. (E) Western blotting of p‐p62 and p62 expression in microglia treated with debris (2 mg/mL) for 24 h after transfection with ACOD1i. (F,G) Densitometric analysis of p‐p62 and p62 expression.

**Figure S5** (A) Synthetic gRNA targeting ACOD1 gene between the exon 2 and 4 was used to carry out ACOD1 gene knockout. (B) F1 mice 2, 3, 4, 8, 9, 15, 17, 18, 20, 22, 29, 31, 32, 33, 34, 35, 36 and 38 were identified by PCR screening.

**Figure S6** (A) The fluorescence quantitative analysis of DCF in Figure 3J. (B) The fluorescence quantitative analysis of DHE in Figure 3J. (C) The fluorescence quantitative analysis of NOX1 in Figure 3K. (D) The fluorescence quantitative analysis of NOX4 in Figure 3K. (E) The fluorescence quantitative analysis of iNOS in Figure 4F. (F) The fluorescence quantitative analysis of iNOS in Figure 4G. (G) The fluorescence quantitative analysis of ACAN in Figure 4J. (H) The fluorescence quantitative analysis of ACAN in Figure 4L.

**Table S1** Prediction of Nrf2 protein binding with seven sites of ACOD1 gene promoters.

**Table S2** The sequence of p62 mutation at Ser 351.

## Data Availability

All data generated or analysed during this study are included in this published article.
