# Supplementary figures and images for: ACOD1, rather than itaconate, facilitates p62‐mediated activation of Nrf2 in microglia post spinal cord contusion
Source: Clin Transl Med. 2024 Apr 22;14(4):e1661. doi: 10.1002/ctm2.1661 (PMC11033726; doi:10.1002/ctm2.1661)

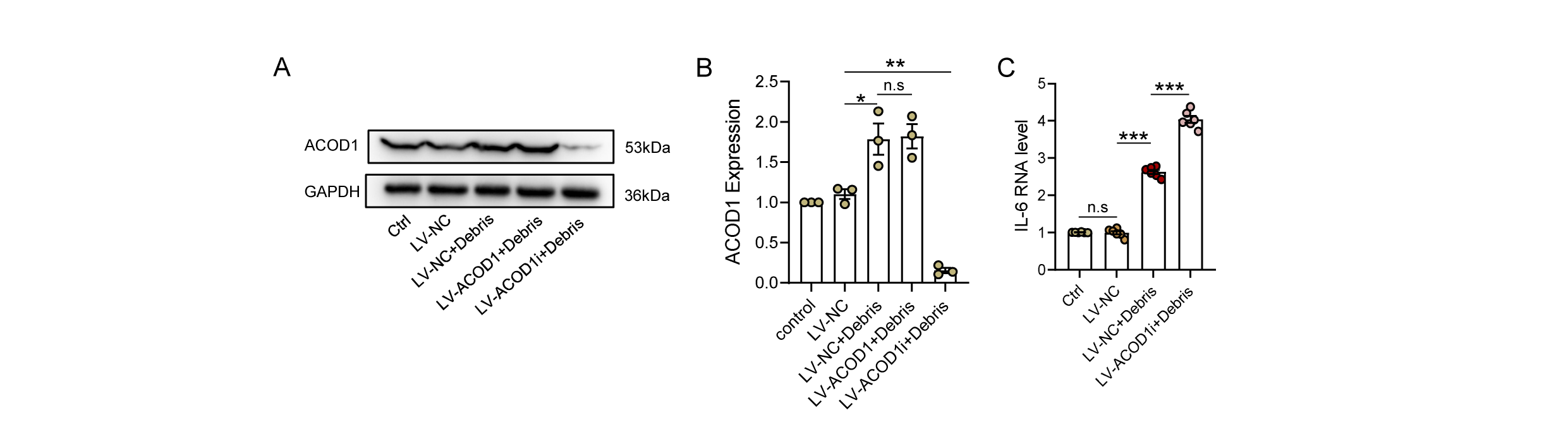

Supplement: Supplementary file 1 — Figure S1 (A) Western blotting for ACOD1 in microglia treated with debris (2 mg/mL) for 24 h after transfection with OE‐ACOD1 or ACOD1i. (B) The densitometry analysis of the ACOD1 expression. (C) Relative mRNA levels of IL‐6 in microglia treated with debris (2 mg/mL) for 24 h after transfection with ACOD1i. [file CTM2-14-e1661-s006.tif]

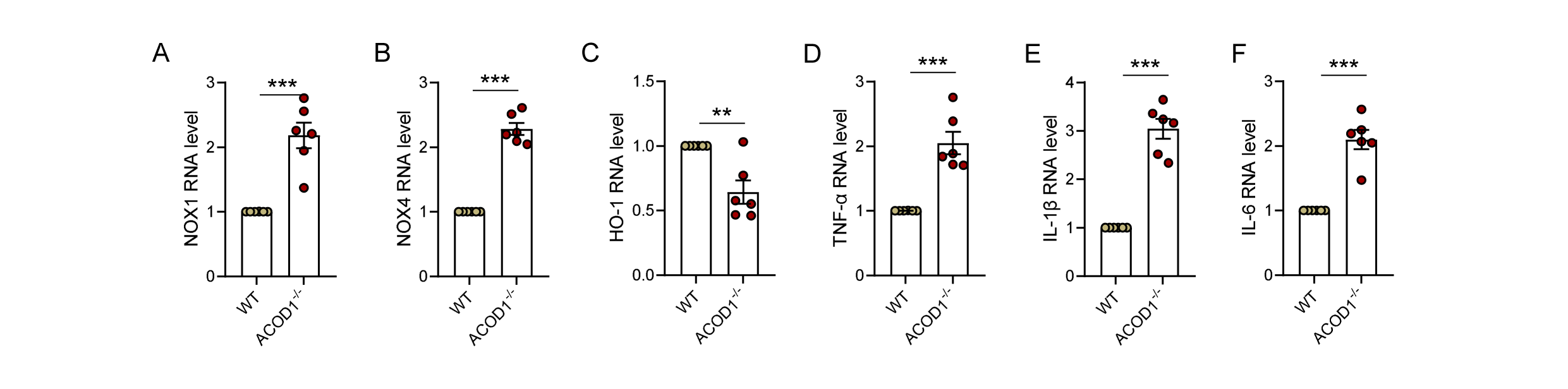

Supplement: Supplementary file 2 — Figure S2 (A) Relative mRNA level of NOX1 in the spinal cord of WT and ACOD1−/− mice at 3 h post‐SCI. (B) Relative mRNA level of NOX4 in the spinal cord of WT and ACOD1−/− mice at 3 h post‐SCI. (C) Relative mRNA level of HO‐1 in the spinal cord of WT and ACOD1−/− mice at 3 hpi. (D) Relative mRNA level of TNF‐α in the spinal cord of WT and ACOD1−/− mice at 3 h post‐SCI. (E) Relative mRNA level of IL‐1β in the spinal cord of WT and ACOD1−/− mice at 3 h post‐SCI. (F) Relative mRNA level of IL‐6 in the spinal cord of WT and ACOD1−/− mice at 3 h post‐SCI. [file CTM2-14-e1661-s007.tif]

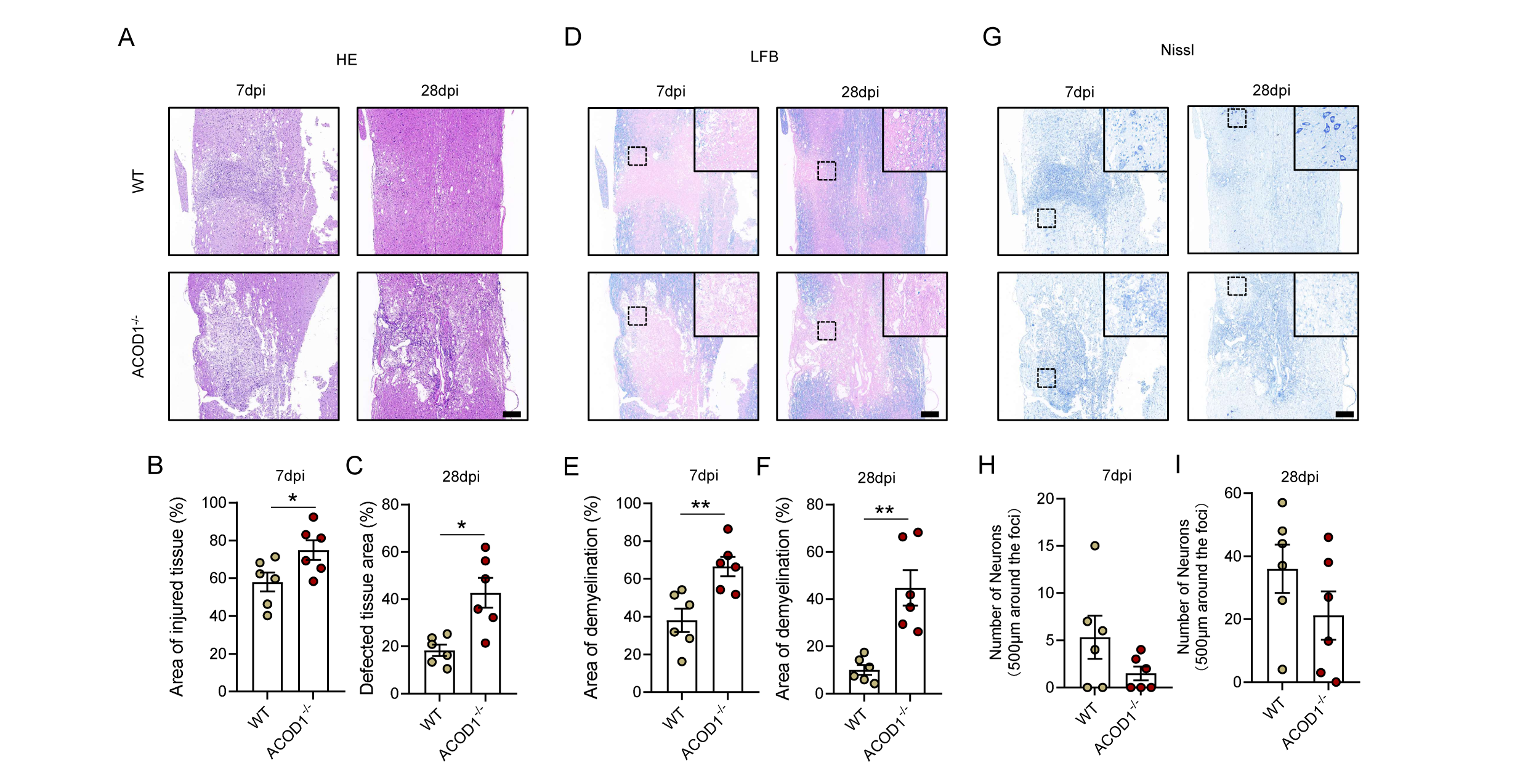

Supplement: Supplementary file 3 — Figure S3 (A) HE staining the injured spinal cord core 3 mm obtained at 7 and 28 days post‐SCI in WT and ACOD1−/− mice; scale bar = 200 μm. (B,C) Quantitative analysis of the affected area at 7 and 28 days post‐SCI. (D) LFB staining images of the injured spinal cord core 3 mm at 7 and 28 days post‐SCI in WT and ACOD1−/− mice; Scale bar = 200 μm. (E and F) Quantitative analysis of the demyelinated area at 7 and 28 days post‐SCI. (G) Nissl staining images of the injured spinal cord core 3 mm at 7 and 28 post‐SCI in WT and ACOD1−/− mice; scale bar = 200 μm. (H,I) Quantitative analysis neuronal survival 7 and 28 days post‐SCI. [file CTM2-14-e1661-s008.tif]

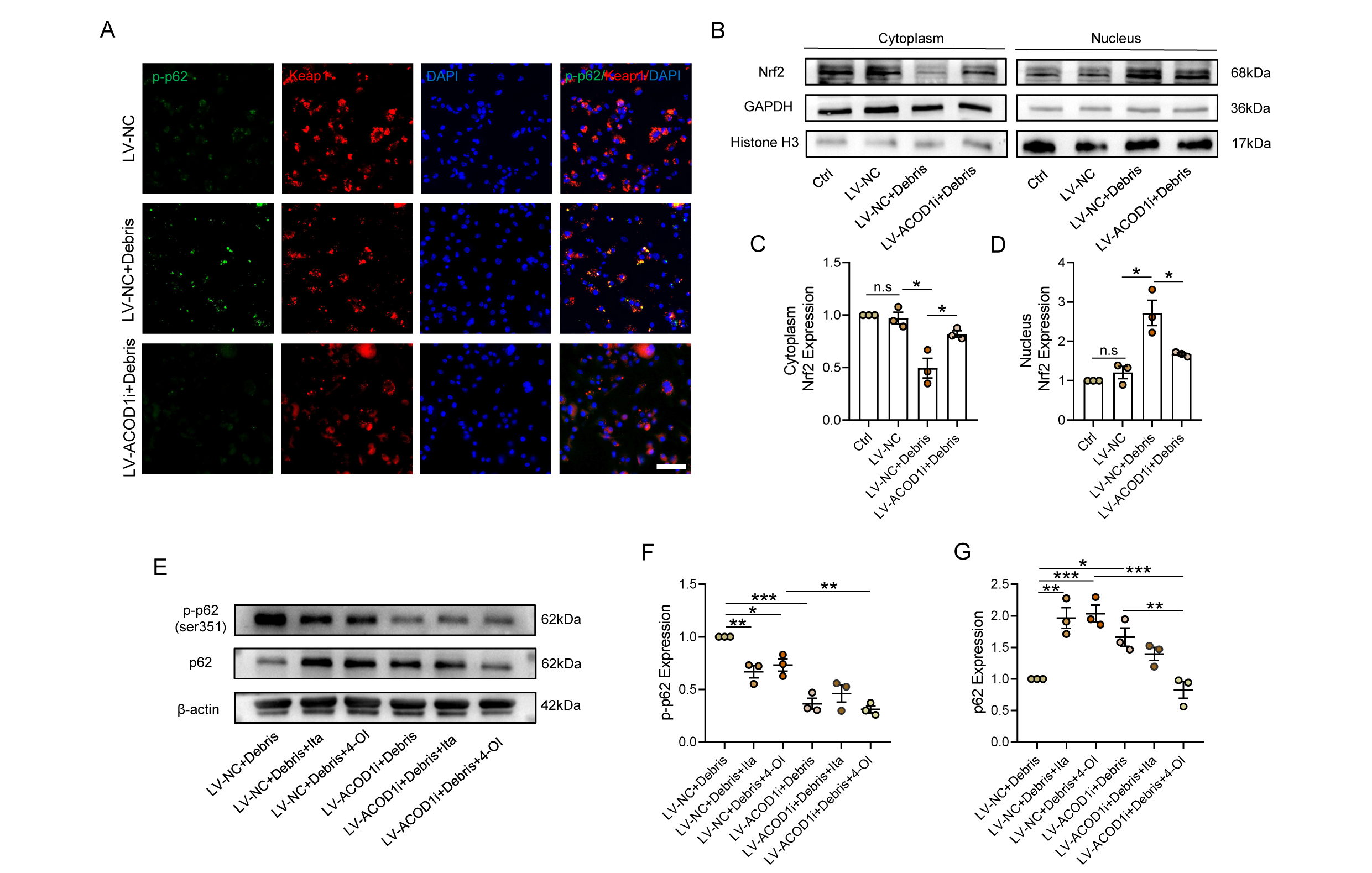

Supplement: Supplementary file 4 — Figure S4 (A) Representative IF labelling images of p‐p62 (green) and Keap1 (red) in microglia treated with debris (2 mg/mL) for 24 h after transfection with ACOD1i; scale bar = 30 μm. (B) Western blotting of Nrf2 in microglia treated with debris (2 mg/mL) for 24 h after transfection with ACOD1i. Histone was used as the control in nuclear. GAPDH was used as the control in cytosol. (C,D) Densitometric analysis of the Nrf2 expression. (E) Western blotting of p‐p62 and p62 expression in microglia treated with debris (2 mg/mL) for 24 h after transfection with ACOD1i. (F,G) Densitometric analysis of p‐p62 and p62 expression. [file CTM2-14-e1661-s004.tif]

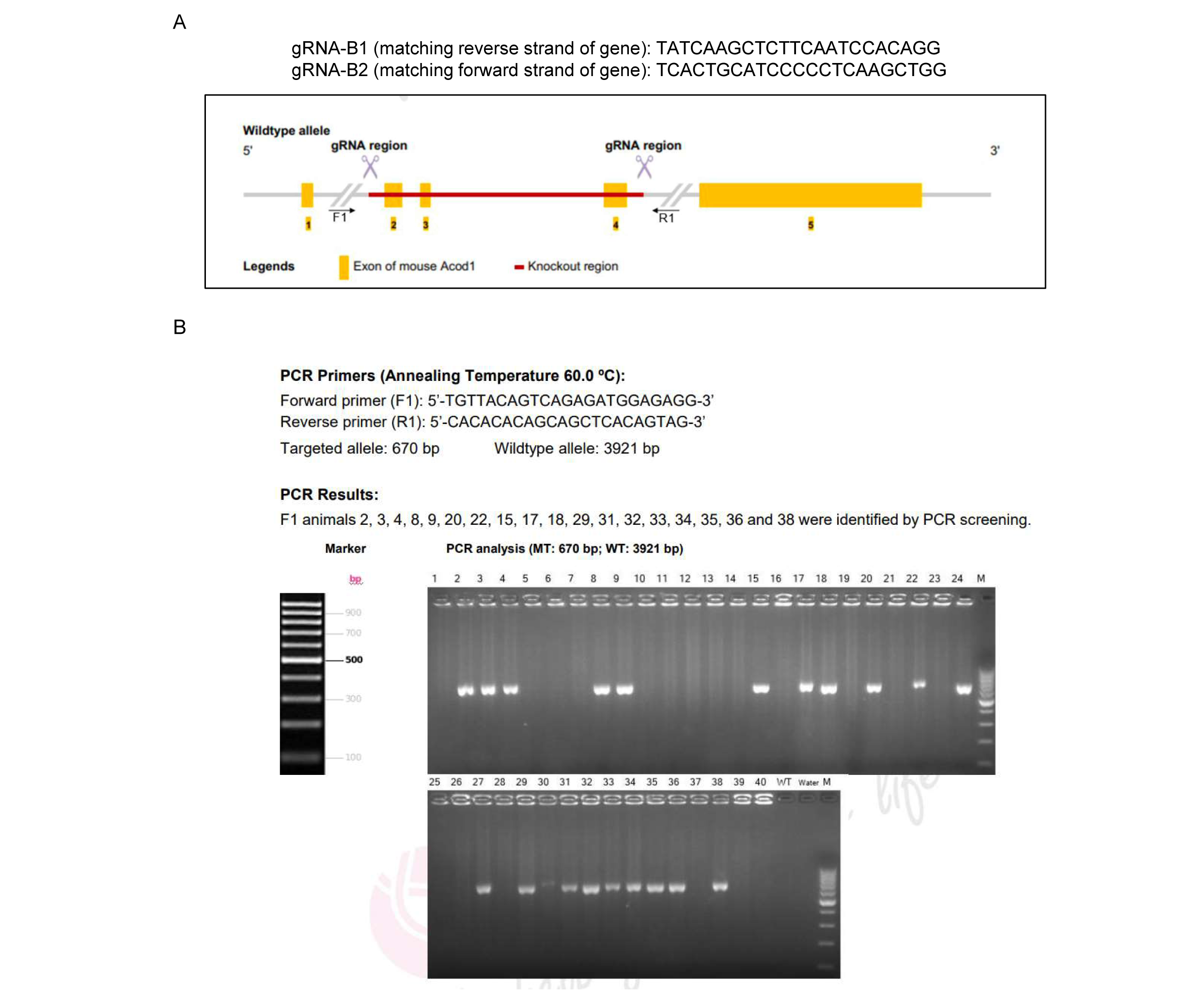

Supplement: Supplementary file 5 — Figure S5 (A) Synthetic gRNA targeting ACOD1 gene between the exon 2 and 4 was used to carry out ACOD1 gene knockout. (B) F1 mice 2, 3, 4, 8, 9, 15, 17, 18, 20, 22, 29, 31, 32, 33, 34, 35, 36 and 38 were identified by PCR screening. [file CTM2-14-e1661-s001.tif]

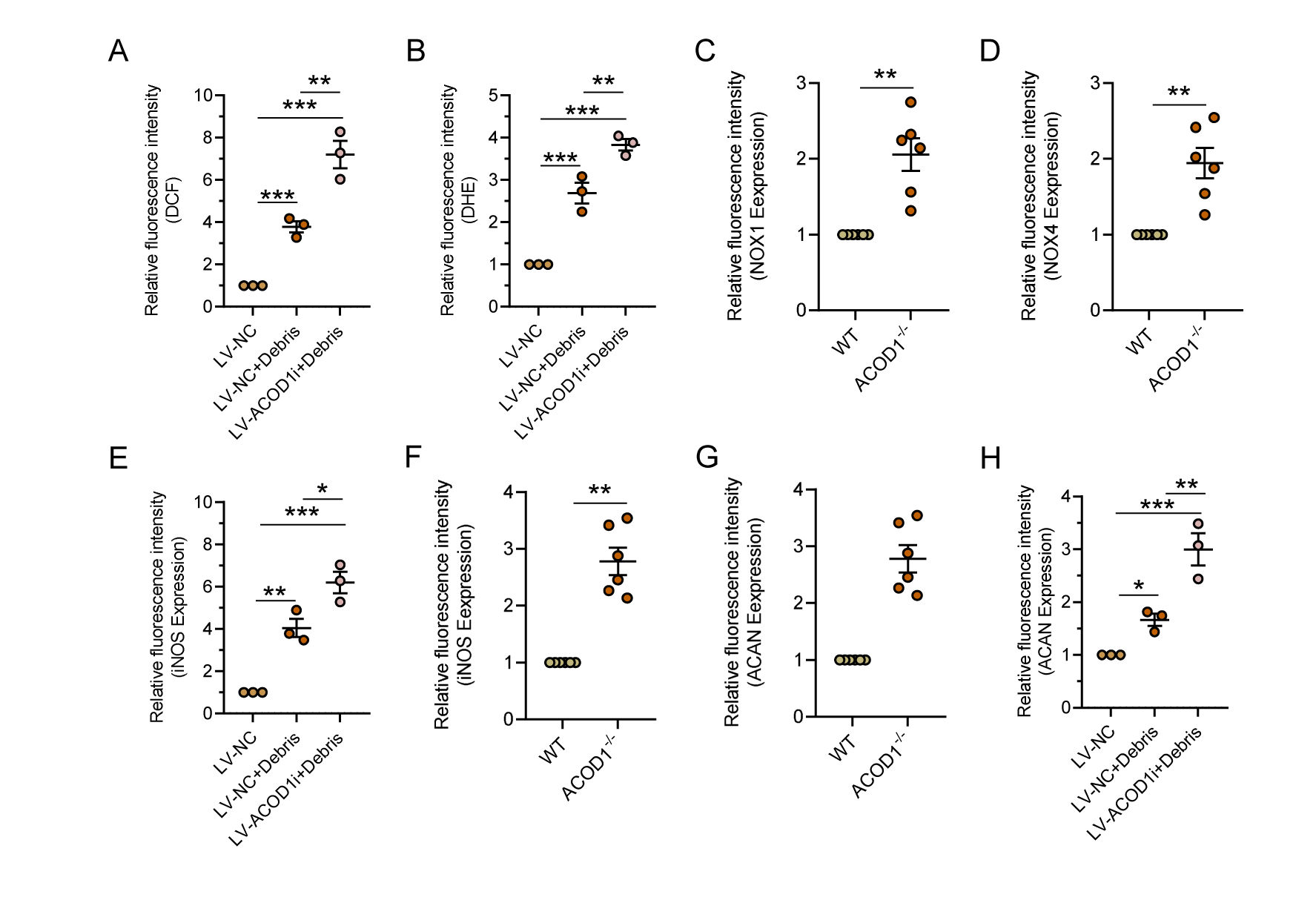

Supplement: Supplementary file 6 — Figure S6 (A) The fluorescence quantitative analysis of DCF in Figure 3J. (B) The fluorescence quantitative analysis of DHE in Figure 3J. (C) The fluorescence quantitative analysis of NOX1 in Figure 3K. (D) The fluorescence quantitative analysis of NOX4 in Figure 3K. (E) The fluorescence quantitative analysis of iNOS in Figure 4F. (F) The fluorescence quantitative analysis of iNOS in Figure 4G. (G) The fluorescence quantitative analysis of ACAN in Figure 4J. (H) The fluorescence quantitative analysis of ACAN in Figure 4L. [file CTM2-14-e1661-s002.tif]
