# Supplementary material for: ACOD1, rather than itaconate, facilitates p62‐mediated activation of Nrf2 in microglia post spinal cord contusion
Source: Clin Transl Med. 2024 Apr 22;14(4):e1661. doi: 10.1002/ctm2.1661 (PMC11033726; doi:10.1002/ctm2.1661)
Supplement: Supplementary file 7 — Table S1 Prediction of Nrf2 protein binding with seven sites of ACOD1 gene promoters. [file CTM2-14-e1661-s003.docx]

**Supplementary Table 1**

**Prediction of Nrf2 protein binding with 7 sites of ACOD1 gene promoters**

Mraked in Grey background is 2000 promoter sequences of **ACOD1** gene.

GATAGTTAATTCTCCGGTATCAGGTTCTTGCACATCTTCCAATAATGAAGTCAGTAGACAGCAAAGGGAAATTTAGAAAACCATATTTGCCACGTTGAAACAATTCAAAGAGGACAGTCAAGACTCAAACACATCCTCTGAGATCCTTGCCAGTGGCAGGATGCCAACCAAGATTCAGTGTCAGGGCTGCAGCTCTATGCTCTCAGCAGTCTTCAGGGTACAAATTAAGAAAGATTGTGGGAGTTACAGTTGCATGTCCTCAACAGGCCTCAGGGTGCCTAGCCCATAAGTTACAAGTGTTACAGCCCTGTGATCTCACCAGGCTTCAGGGGGACACACAAAAACTGTTGGTAAGTATTACAGTTTGAACGCTGTTAGGGGCTTTGGAGTGCTAGCCAATAGCAATCCCTGGCAGAACAATTCTGCAGAGTGATCATTGACAGGGGTAACAAGCCTAGCATACCTCCTGTCTCTGGCAGGTCTTGAAGGCTTGAGTTGCAAATCTCTGCAAGTCTCTGAAGCAATGAATCTTGGGTAGTCCAAGTAGTTGCATGAATGAATTACCTTCTGAATTCAAAATATGAAGCTTTGTGGACTGAAGGAAGATGAGTCCCCCAACCTGCAGCTGCAGAAAGAGCCTGTAGACTGCTCTGAGCACATTCTCTGCCCGGGACAGAAGCCTCCAGTGTGGCAAGACTCTTAACTAGTCTGTCTCCTCTCTGAAAACTTAAAGGCATTCTTCTTTCTCAGGAGAAAGTCTGGGAGCCAAGCTCCTGGTCAGACAGGGATCCAGCTGAGAGCAGGACTTCATCTTTAGGCCTCTTCTTCTGCAAAAAAGGGGAACATGTGAGGGCCAGGACACAGGTCAGAACCAGAAGACTTCAGAGAGTCAGAATCCTGCCTGCCTGGGTGCCTGGTTCTGAAGTCCTCCTCCTCCTCCTCCTCCTCCTCCTCCTCCTCCTCCTCCTCCTCCTCTTCCTCCTCCTCCTTCTTTCTCTCTCTCTCTCTCTCTCTCTCTCTCTCTCTCTCTCTCTCTCTCTCTCTCTCTCTCTCTCTCTTCTCCAAGGAATAATCTGTGAAGGATGATTTTCCAGAGTTCTCCTCTATTACACTCAACCAGGTGGTCTCTGATTAGCCAAATGTGTGTACTTGGCATGTGTGTGTGTGTGTGTGTGTGTGTGTGTGTGTGTGTGTGTGTACATGTGTCTGTGTGGTGTGTATTTAGATATTTTTAATTAATTTTTTGAGATTTTAATTTATTTATTTATGTATTTACAACATTTCCACCCCTCCTCCTTCTCTTCTTTTAACTCTGGCCACCATTCCTCACAAATTCATGGCCTCTTCTTTAGTTATCACACACACACACACACACACACACACACACACACACGTGTGCACACATATGTACACGTACTTTCTCAACTGTCTCTGAGCCTTAGGTGTAGGGGTTTCATTGTTGGTATATCAGTTAGGTCTGGGCACCCATGCTCACTTACTCGTTATAGATAGTTGTAGTAGTCGTCTCCATCTACAATAAAAAGAAGCATCGTTGATGAGGGGCAGAGCTACACTTATCTGTGCGTGAGTAAGTATTTAGAATGCAGCTAGGTTTAGGGAACTGGCAGTGGTAGGCTCTCCTCTAGGATTCATAACCTTTAGGTTATGGCACATTTAGTGACTTCGTTAGTTACCTCTGGGTTATCGCCTCTGAAATTATGATGTGATTGGTGGCTTTGTTCACAGTACCAGTCATGGATTTCCTTCTGGTAAGCAGGCTCTAAGTCCAATAAGAACCAGTCAGGGTTTTTGTTTTTTGTTTTTTTCATCATTCCCAGAAGCTGTTTCATGGTTGGTTGGTGACAATGGGCTGTCTGTGAGAAGGCTGCTCTGAGTGACTCCTTGCCTTTCCAGATCTGGGGGCTTTTCTTCCTCTGTCATAGTCTTGCTATTTCCTTGTGATAAGTCTTCAAGAGTGGGATGCCCAGTAAAGAGAGTGTGCACTCTTCTCACCTATGGCCCCTAGCTGGGACAGGCCATCCCTCTGTAATTATAACTTAGTTTTTCTTATGTCTGTGAGGAATCATCAGATTATGGCCGCTGTAACACCTCCTCTCATCAGCCCTACATCTATAAGGGAGCTCTACACAACACAACAAAGGAAGTTTAAGTGAGGTCATTGGGGAGGTGTTGAAATGGTGGAAATTCCATGGTTGAAACAAAAGTGAAAGGGACAGACCTGGAGGGAGTGACTGTGTATAAAGGCACACGTCCACTAAAGGCCCCCAGCCAGCAACTACTCCTGCCATCCACTCCTGAGCCAGTTACCCTCCAGAGCAACATGATGCTCAAGGTATCGTAGCACTTGATGGCGCTTGCCTAAACTGCCTTCCTGGCTTGATTCTTCCTTTCTCAATCAGTTGGTCTTTTGCTAGCTATCTGCTCTGCTGCAGAAAGGACATCTTAGCTTTGCTTATGGTCTCTGAGCTGCCTGATTTAGCTATCTGTTGGCTTTCATCAGTATTTGACAGACAATGCCCATGATCCCATTGCTTCCTCCTAGCAAGAGAGTTAGGCCTGGTAAGTTCACTGAACCTCCACCTCTTGGTTTTGTGTGTGTCCTCTGTGGGACAGGAGCACATCTGTGATGGGAGCACAGGGGCACATCTGTAATTATCTATTTGCTCTCATGTACCCTGTAGTCCCCTGGCAGGTTTTTTAAGTTGGATGGCCTCATTTACTGGTTTGGCAGTTTAGAGAACAGTGCACAGGGAACTTGCCAGGGCTCCTGACACTTATTATAAAACGAATGACTCCATACTCCGCTTTAATCTATACATTAGTCACAGGAGCTTACTAAAAAGATAGACAAGTGCCCCTATAAAGTCAAGCACGCCAGCAAGACATTTGACTTGTAGATGAAGGATGACTCACAGGAATCTGAGTGGAGCTATTGTCCCCCTTATGATCTCTCCCTCCATGGTCAGGGAGGGCGGCTTGTTCTTTGGCTCTCAGATCGTGTTCAGCACTATCAGGTCAGGGGAGCCCAGTGGTCTGTCGTAAATCACATAAGCAGTGCCAGCCCGATGCCTTGAGATCTATGCTGCTGTTCCTCAT

Primers of binding sites 1 and 2 correspond successively to the red marks.

Primers of binding sites 3 to 7 correspond successively to the yellow marks.

**Primers list**

| Gene Name | Forward Sequence (5’-3’) | Reverse Sequence (5’-3’) |
| --- | --- | --- |
| Binding site 1 | ATCTCACCAGGCTTCAGG | ATGCTAGGCTTGTTACCC |
| Binding site 2 | TGGTAAGCAGGCTCTAAG | TCTGGAAAGGCAAGGAGT |
| Binding site 3 | GTCTTGAAGGCTTGAGTT | GCAGTCTACAGGCTCTTT |
| Binding site 4 | CTCCTCCTCCTTCTTTCT | CACCTGGTTGAGTGTAAT |
| Binding site 5 | CTTATCTGTGCGTGAGTA | CTAAATGTGCCATAACCT |
| Binding site 6 | GGGATGCCCAGTAAAGAG | TTACAGCGGCCATAATCT |
| Binding site 7 | GGTGTTGAAATGGTGGAA | TGGATGGCAGGAGTAGTT |
