# Supplementary material for: ACOD1, rather than itaconate, facilitates p62‐mediated activation of Nrf2 in microglia post spinal cord contusion
Source: Clin Transl Med. 2024 Apr 22;14(4):e1661. doi: 10.1002/ctm2.1661 (PMC11033726; doi:10.1002/ctm2.1661)
Supplement: Supplementary file 8 — Table S2 The sequence of p62 mutation at Ser 351. [file CTM2-14-e1661-s005.docx]

小鼠 P62 NM_011018

P62 NM_011018(S351A)

NM_011018(S351A)

ATGGCGTCGTTCACGGTGAAGGCCTATCTTCTGGGCAAGGAGGAGGCGACCCGCGAGATCCGCCGCTTCAGCTTCTGCTTCAGCCCGGAGCCGGAGGCGGAAGCCCAAGCCGCGGCCGGCCCGGGGCCCTGCGAGAGGCTGCTGAGCCGAGTGGCTGTGCTGTTCCCCACGCTGAGGCCTGGCGGCTTCCAGGCGCACTACCGCGATGAGGATGGGGACTTGGTTGCCTTTTCCAGTGATGAGGAGCTGACAATGGCTATGTCCTATGTGAAAGATGACATCTTCCGCATCTACATTAAAGAGAAGAAGGAGTGCCGGCGGGAACATCGCCCACCATGTGCTCAGGAGGCACCCCGAAACATGGTGCACCCCAATGTGATCTGTGATGGTTGCAACGGGCCTGTGGTGGGAACTCGCTATAAGTGCAGTGTGTGCCCAGACTACGACCTGTGCAGCGTGTGCGAGGGGAAGGGCCTGCACAGGGAACACAGCAAGCTCATCTTTCCCAACCCCTTTGGCCACCTCTCTGATAGCTTCTCTCATAGCCGCTGGCTTCGGAAGCTGAAACATGGACACTTTGGCTGGCCTGGCTGGGAGATGGGCCCACCGGGGAACTGGAGCCCACGTCCTCCTCGTGCAGGGGATGGCCGCCCTTGCCCTACAGCTGAGTCAGCTTCTGCTCCACCAGAAGATCCCAATGTCAATTTCCTGAAGAATGTGGGGGAGAGTGTGGCAGCTGCCCTCAGCCCTCTAGGCATTGAGGTTGACATTGATGTGGAACATGGAGGGAAGAGAAGCCGCCTGACACCCACTACCCCAGAAAGTTCCAGCACAGGCACAGAAGACAAGAGTAACACTCAGCCAAGCAGCTGCTCTTCGGAAGTCAGCAAACCTGACGGGGCTGGGGAGGGCCCTGCTCAGTCTCTGACAGAGCAAATGAAAAAGATAGCCTTGGAGTCGGTGGGACAGCCAGAGGAACAGATGGAGTCGGGAAACTGCTCAGGAGGAGACGATGACTGGACACATTTGTCTTCAAAAGAAGTGGACCCAGCTACAGGTGAACTCCAGTCTCTACAGATGCCAGAATCGGAAGGGCCAAGCTCTCTAGACCCCTCACAGGAAGGACCCACAGGGCTGAAGGAAGCTGCCCTATACCCACATCTCCCACCAGAGGCTGATCCCCGGCTGATTGAGTCCCTCTCCCAGATGCTGTCCATGGGTTTCTCGGATGAAGGCGGCTGGCTCACCAGGCTCCTACAGACCAAGAATTACGACATCGGGGCTGCTCTGGACACGATCCAGTATTCGAAGCACCCTCCACCATTGTGA
